# *In Situ* Anodically Oxidized BMIm-BF_4_: A Safe and Recyclable BF_3_ Source

**DOI:** 10.1021/acs.joc.1c00932

**Published:** 2021-07-02

**Authors:** Martina Bortolami, Leonardo Mattiello, Vincenzo Scarano, Fabrizio Vetica, Marta Feroci

**Affiliations:** † Department of Basic and Applied Sciences for Engineering (SBAI), Sapienza University of Rome, via Castro Laurenziano, 7, 00161 Rome, Italy; ‡ Department of Chemistry, Sapienza University of Rome, piazzale Aldo Moro, 5, 00185 Rome, Italy

## Abstract

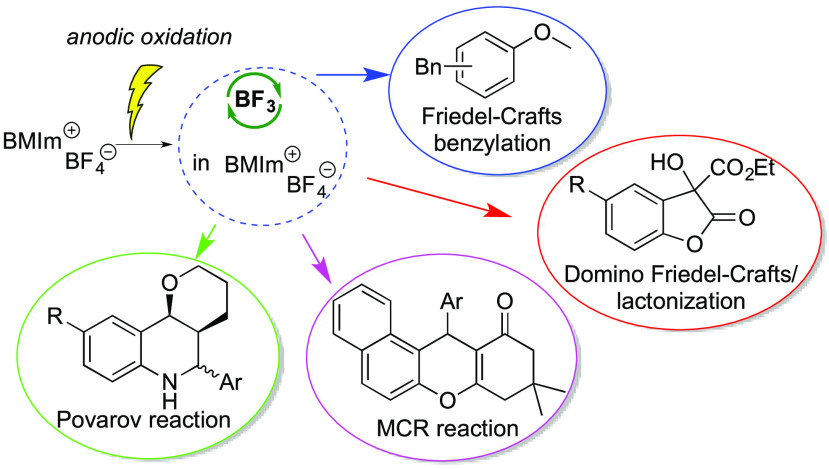

The anodic oxidation of 1-butyl-3-methylimidazolium tetrafluoroborate (BMIm-BF_4_) efficiently generates BF_3_ from BF_4_^–^. This Lewis acid, strongly bound to the ionic liquids, can be efficiently used in classical BF_3_-catalyzed reactions. We demonstrated the BF_3_/BMIm-BF_4_ reactivity in four reactions, namely, a domino Friedel–Crafts/lactonization of phenols, the Povarov reaction, the Friedel–Crafts benzylation of anisole, and the multicomponent synthesis of tetrahydro-11*H*-benzo[*a*]xanthen-11-ones. In comparison with literature data using BF_3_-Et_2_O in organic solvents, in all the presented cases, analogous or improved results were obtained. Moreover, the noteworthy advantages of the developed method are the *in situ* generation of BF_3_ (no storing necessity) in the required amount, using only the electron as redox reagent, and the recycling of BMIm-BF_4_ for multiple subsequent runs.

Boron trifluoride is a well-known Lewis acid, often used in organic synthesis to carry out many acid-catalyzed transformations.^1^

Although this reagent is very common, its use may face problems and small accidents due to its high reactivity and volatility. Additionally, this gas is highly toxic and corrosive and has a suffocating odor.^2^

To make BF_3_ easier to handle, liquid etherate complexes, consisting of a 1:1 molar ratio of BF_3_ and ether (usually dimethyl or diethyl), are used and dissociated under appropriate temperature and pressure conditions.^3^

Nonetheless, these compounds show corrosive properties and flammability, so it is necessary to use them under a hood, wearing nitrile gloves and eye protection.^4^ Moreover, they are sensitive to humidity and form acidic fumes in moist air.

The *in situ* generation of BF_3_ in the exact amount needed minimizes these problems.

Organic electrochemistry can help with this scope.^5^ In fact, BF_3_ can be easily obtained by anodic oxidation of the BF_4_^–^ anion (Scheme 1).^6^

**Scheme 1:**
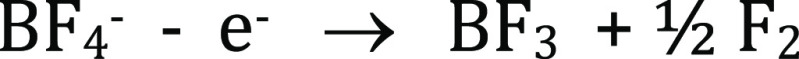
BF_3_ Anodic Generation

When using electrochemistry, the reagent is the electron (inherently nonpolluting and cheap), very easy to dose simply by closing or opening the electrical circuit.

ILs are liquid salts formed by a large, nonsymmetrical organic cation and (usually) a noncoordinating anion (organic or inorganic).^7^ Their use as solvents in organic transformations is growing in the past years, due to their ability to solubilize organic and inorganic compounds and, mainly, to their virtually null volatility, allowing for their easy recovery.^8^ In organic electrochemistry, they can be used as supporting electrolytes or also as solvents, permitting carrying out electrolyses and, after workup, to recover the IL.^9^ In this context, the most frequently used class of ILs is the imidazolium one, which are cheap, liquid in a wide range of temperatures, and possess good solvating properties. Nevertheless, imidazolium ILs are in some cases reactive under electrochemical conditions.^10^ In fact, the cathodic limit of an imidazolium IL (unsubstituted at the 2-position) is usually the C2–H bond scission with formation of the corresponding N-heterocyclic carbene (NHC), widely exploited,^6,11^ while the anodic limit is the oxidation of the anion. In the case of 1-butyl-3-methylimidazolium tetrafluoroborate (BMIm-BF_4_), the oxidation of the anion forms BF_3_,^6^ as previously stated (Scheme 1). The relatively high potential for BF_3_ generation prevents the presence of electroactive substrates in solution during electrolysis (see cyclic voltammetries in the Supporting Information). We were interested in an alternative, less dangerous source of BF_3_, generated *in situ* and thus not stored. The electrochemical oxidation of BF_4_^–^ in IL seemed the good choice, and we carried out some classical BF_3_-catalyzed reactions in anodically oxidized BMIm-BF_4_, being this IL really easy to recycle after ethereal extraction. In order to avoid interferences from the cathodically generated NHC, a divided cell was used.

The advantages in this BF_3_ source can be summarized in

- *in situ* generation, avoiding the storage (simple galvanostatic electrolysis)
- easy to dose (current on/off)
- no fumes production (strong interaction with IL)
- no particular sensitivity to moisture (IL as moist protector)
- easy IL recovery and multiple recycling after ethereal extraction

The main disadvantage derives from the use of the IL, i.e., the low solubility of apolar molecules.

The examples considered (Scheme 2) are intended to demonstrate the efficiency of this system in classical BF_3_-catalyzed reactions, and thus no extensive studies for the optimization of yields and reaction scope are reported. It should be underlined that, when more than one reaction was carried out on a particular substrate, the same IL was used in all reactions, recycled after ethereal extraction and submitted to a new anodic oxidation. To the best of our knowledge, anodically generated BF_3_ in IL was used only in one paper^12^ reporting the BF_3_ induced Michael addition of a 1,3-dicarbonyl compound to methyl vinyl ketone, without IL recycling.

**Scheme 2:**
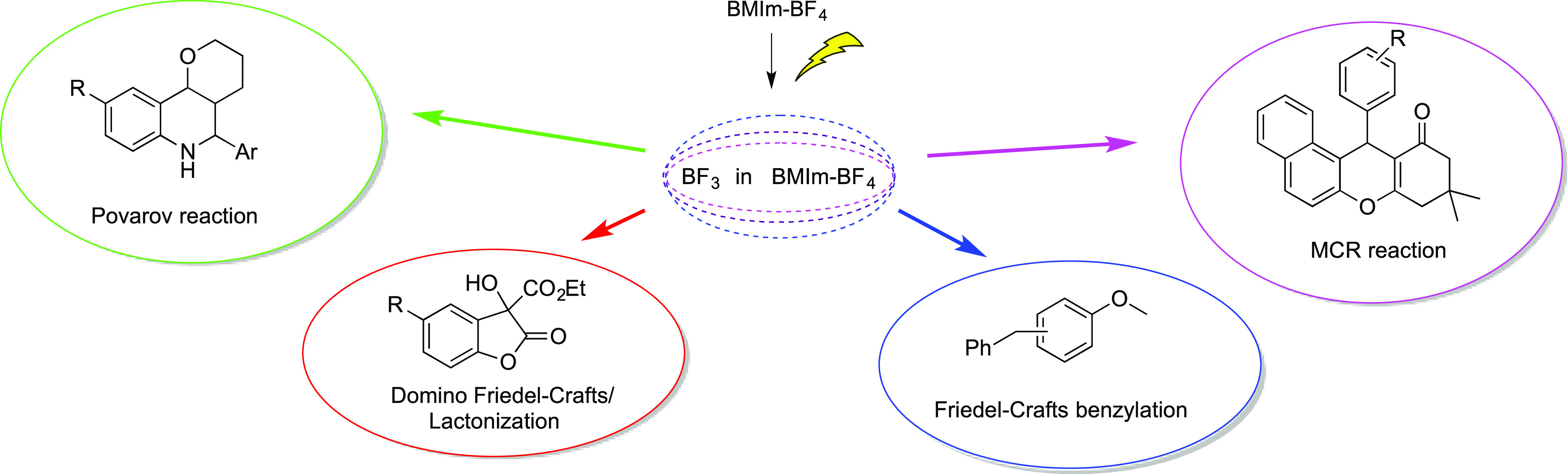
Exploited BF_3_-Catalyzed Reactions

The reaction between a phenol **1** and diethyl ketomalonate **2**, in the presence of a Lewis acid, leads to the formation of a 3-hydroxybenzofuran-2-one **3** and, in the case of incomplete reaction, of the 2-substituted phenol **4** (Table 1).^13^ These products derive from a Friedel–Crafts phenol alkylation in the 2-position, followed by a cyclization with ethanol elimination. The increase of the temperature to 60 °C promoted the lactonization, giving selectively the 3-hydroxybenzofuran-2-one **3**.

**Table 1:**
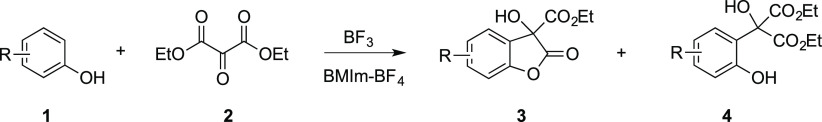
BF_3_-Catalyzed Reaction between Phenols and Diethyl Ketomalonate^a^

entry	R	BF_3_ (%)^b^	*T*/time	**3**^c^	**4**^c^
1^d^	4-OCH_3_ (**1a**)	100	r.t./15 h	57% (**3a**)	17% (**4a**)
2^d^	4-OCH_3_ (**1a**)	100	50 °C/4 h	63% (**3a**)	
3^d^	4-OCH_3_ (**1a**)	30	r.t./24 h	56% (**3a**)	19% (**4a**)
4^d^	4-OCH_3_ (**1a**)	30	r.t./2 h, 50 °C/2 h	32% (**3a**)	17% (**4a**)
5^d^	4-OCH_3_ (**1a**)	30	50 °C/4 h	79% (**3a**)	
6	H (**1b**)	30	50 °C/4 h	88% (**3b**)	
7	fused Ph (2-naphthol, **1c**)	30	50 °C/4 h	86% (**3c**)	
8, lit.^13^	4-OCH_3_ (**1a**)	30, BF_3_-Et_2_O in CH_2_Cl_2_	r.t./24 h	36% (**3a**)	traces
9, lit.^13^	4-OCH_3_ (**1a**)	TiCl_4_, 10% in CHCl_3_	60 °C/6 h	84% (**3a**)	traces
10, lit.^13^	H (**1b**)	TiCl_4_, 10% in CHCl_3_	60 °C/6 h	87% (**3b**)	
11, lit.^13^	fused Ph (2-naphthol, **1c**)	TiCl_4_, 10% in CHCl_3_	r.t./2 h	95% (**3c**)	

a BMIm-BF_4_ (divided cell) was electrolyzed (galvanostatic conditions: 10 mA cm^–2^) on platinum electrodes (r.t., N_2_). At the end of electrolysis, phenol **1** (0.5 mmol) and diethyl ketomalonate **2** (0.5 mmol) were added to the anolyte. The mixture was stirred (*T* and time in table) and then extracted with diethyl ether.

b Amount of electrogenerated BF_3_ with respect to starting phenol, admitting a 100% current efficiency (96.5 C: 1 mmol of BF_3_).

c Isolated yields after column chromatography.

d Entries 1–5: the same recycled IL was used.

Different Lewis acids in catalytic amounts in CH_2_Cl_2_ at room temperature were used, with good yields.^13^

We tested the anodically generated BF_3_ in BMIm-BF_4_ in this reaction, and the results are reported in Table 1, along with the corresponding literature data, for a useful comparison.

As reported in Table 1, high yields in products **3a**–**c** (entries 5–7) were obtained using a 30% maximum of catalyst (calculated admitting a 100% current yield), comparable with those obtained in the literature using the best experimental conditions, i.e., TiCl_4_ as Lewis acid (entries 9–11). A direct comparison with literature data can be made considering entries 3 and 8, in which the same phenol (**1a**), amount of BF_3_ (30%), reaction time and temperature were used. **3a** was obtained in 56% in IL (with a 19% of intermediate **4a**) with respect to 36% of **3a** obtained in CH_2_Cl_2_. Also in this case, BMIm-BF_4_ demonstrated to be a solvent suitable for reactions involving dipolar intermediates.^11d^ Additionally, from the high yield using 30% of BF_3_, we can infer that the IL acts as an efficient solvent to bind this volatile reagent and ensures the reiteration of the catalytic cycle. Moreover, the eco-friendly character of this reaction in IL is demonstrated not only by the use of electricity to generate the catalyst but also by the use of the same IL sample in five subsequent runs (entries 1–5), without reactivity loss.

The second reaction considered is the hetero-Diels–Alder Povarov reaction.^14^ It is the reaction between an aryl amine **5**, an aryl aldehyde **6** (with formation of the corresponding electron-poor imine), and an electron-rich dienophile (usually 3,4-dihydro-2*H*-pyran **7** or 2,3-dihydrofuran), yielding the corresponding tetrahydroquinoline **8** in a *cis*/*trans* diastereomeric mixture (Table 2).

**Table 2:**
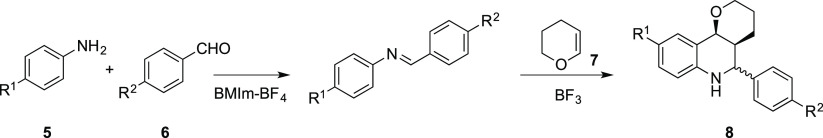
BF_3_-Catalyzed Povarov Reaction^a^

entry	R^1^, R^2^	**5**/**6**/**7**^b^	BF_3_ (%)^c^	**8**^d^	*cis*/*trans*^e^
1^f^	CH_3_/H	1/1/4	50	68% (**8a**)	76/24
2^f^	CH_3_/H	1/1/3	25	96% (**8a**)	71/29
3^f^	CH_3_/H	1/1/3	50	91% (**8a**)	79/21
4^f^	CH_3_/H	1/1/2	50	82% (**8a**)	68/32
5^f^	CH_3_/H	1/1/1	50	37% (**8a**)	71/29
6^g^	OCH_3_/H	1/1/3	50	79% (**8b**)	76/24
7^g^	OCH_3_/H	1/1/3	25	89% (**8b**)	92/8
8^h^	CH_3_/OCH_3_	1/1/3	50	69% (**8c**)	65/35
9^h^	CH_3_/OCH_3_	1/1/3	25	63% (**8c**)	71/29
10, lit.^14^	H/H	1/1/1	3, BF_3_-Et_2_O/Et_2_O	15%	
11, lit.^16^	OCH_3_/H	1/1/2	30, I_2_/MeCN	95% (**8b**)	8/92

a Aniline **5** (0.5 mmol), benzaldehyde **6** (0.5 mmol), and 3,4-dihydro-2*H*-pyran **7** (amount as in table) were added to the anodically generated BF_3_/BMIm-BF_4_ (footnote *a* of Table 1). The mixture was stirred at r.t. for 3 h and then extracted with diethyl ether.

b **5** to **6** to **7**, molar ratio.

c Amount of electrogenerated BF_3_ with respect to starting aniline, admitting a 100% current efficiency (96.5 C: 1 mmol of BF_3_).

d Isolated yields after column chromatography.

e Determined by the ^1^H NMR of the crude.

f Entries 1–5: the same recycled IL was used.

g Entries 6 and 7: the same recycled IL was used.

h Entries 8 and 9: the same recycled IL was used.

We tested the electrogenerated BF_3_/BMIm-BF_4_ system in this reaction, the imine being obtained in quantitative yield by simple addition of aniline **5** and benzaldehyde **6** to the IL (a noteworthy dehydrating agent). As reported in Table 2, this reaction works well using a theoretical 25% amount of BF_3_ (with respect to the imine), in the presence of 3 equiv of dihydropyran **7**. High yields of compounds **8a**–**c** were obtained (96%, 89%, and 69% yields, entries 2, 7, and 8, respectively). In all the cases in this work, the yields obtained are higher when compared with analogous literature data (entry 10).^14^ Moreover, the *cis* isomer was synthesized preferentially, in accordance with other methodologies which employ Lewis acids in classical organic solvents^15^ and with opposite diastereoselectivity observed by using I_2_ as catalyst (Table 2, entry 11).^16^ Also in this case, it was possible to reuse the same ionic liquid (entries 1–5, Table 2) in subsequent runs without reactivity loss.

The third reaction considered is the Friedel–Crafts benzylation of anisole **10** with benzyl alcohol **9** (Table 3), in which anisole is monobenzylated in the *ortho* or *para* positions (the *meta* isomer being present only in traces).^17^

**Table 3:**
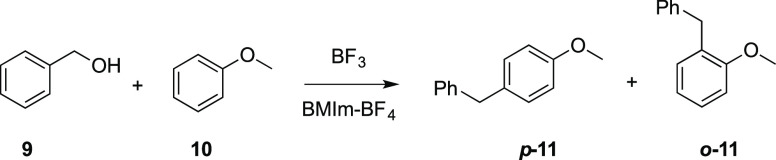
BF_3_-Catalyzed Friedel–Crafts Benzylation of Anisole^a^

entry	**9**/**10**^b^	BF_3_ (%)^c^	**11** (%)^d^	**11**, *p*/*o*^e^
1^f^	1/2	100	18	53/47
2^f^	1/3	100	60	57/43
3^f^	1/4	100	69	54/46
4^f^	1/4	50	16	58/42
5^f^	1/4	150	80	58/42
6, lit.^17c^	1/18^g^	120, BF_3_-Et_2_O/H_2_O, 80 °C	61	>99/1
7, lit.^18^	1/4	30, Yb(OTf)_3_/BMIm-OTf, 65 °C	71	57/43

a Anisole **10** (amount as in table) and benzyl alcohol **9** (0.5 mmol) were added to the anodically generated BF_3_/BMIm-BF_4_ (footnote *a* of Table 1). The mixture was stirred at r.t. for 4 h and then extracted with diethyl ether.

b **9** to **10**, molar ratio.

c Amount of electrogenerated BF_3_ with respect to starting **9**, admitting a 100% current efficiency (96.5 C: 1 mmol of BF_3_).

d Isolated yields after column chromatography.

e Determined by the ^1^H NMR of the crude.

f Entries 1–5: the same recycled IL was used.

g 2,4-Dichlorobenzyl alcohol was used as benzylating agent.

Good yields in benzylated anisole **11** were obtained using a stoichiometric (69%, entry 3) or overstoichiometric (80%, entry 5) amount of catalyst. Moreover, milder reaction conditions were used, with respect to the literature (r.t. vs 65–80 °C, Table 3), and more importantly, the efficient recycling of the IL was demonstrated (entries 1–5).

The literature data here reported for comparison (entry 6, Table 3) showed that the thermodynamic favorite product *p***-11** can be obtained using a very large excess of anisole (**10** to **9**: 18/1) at 80 °C. The positive effect of an imidazolium IL as solvent in this reaction, involving charged species as intermediates, is confirmed by literature data, besides the results obtained in this work (Table 3, entry 7).^18^

The last example is the multicomponent synthesis of tetrahydro-11*H*-benzo[*a*]xanthen-1-one **13** from benzaldehyde **6**, 2-naphthol **1c**, and dimedone **12** (Table 4).

**Table 4:**
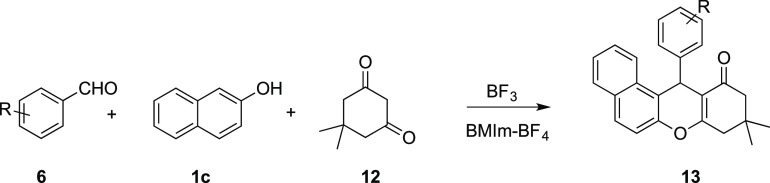
BF_3_-Catalyzed Synthesis of Substituted Tetrahydro-11*H*-benzo[*a*]xanthen-11-ones^a^

entry	R	BF_3_ (%),^b^*T* (°C), *t* (h)	**13**^c^
1^d^	H	25, r.t., 3 h	68% (**13a**)
2^d^	H	25, 60 °C, 1 h	85% (**13a**)
3^d^	H	25, 60 °C, 2 h	87% (**13a**)
4^e^	4-Cl	25, 60 °C, 1 h	67% (**13b**)
5^e^	4-Cl	25, 60 °C, 2 h	76% (**13b**)
6, lit.^19^	H	20, BF_3_-Et_2_O/EtOH, 80 °C, 45 min	82% (**13a**)
7, lit.^19^	4-Cl	20, BF_3_-Et_2_O/EtOH, 80 °C, 45 min	80% (**13b**)

a 2-Naphthol **1c** (0.5 mmol), benzaldehyde **6** (0.5 mmol), and dimedone **12** (0.5 mmol) were added to the anodically generated BF_3_/BMIm-BF_4_ (footnote *a* of Table 1). The mixture was stirred (time and temperature as in table) and then extracted with diethyl ether.

b Amount of electrogenerated BF_3_ with respect to starting 2-naphthol, admitting a 100% current efficiency (96.5 C: 1 mmol of BF_3_).

c Isolated yields after column chromatography.

d Entries 1–3: the same recycled IL was used.

e Entries 4 and 5: the same recycled IL was used.

The literature reaction was carried out in boiling ethanol (80 °C) with 20% of BF_3_-Et_2_O, obtaining high yields of 9,9-dimethyl-12-aryl-8,9,10,12-tetrahydro-11*H*-benzo[*a*]xanthen-11-ones **13** (Table 4, entries 6 and 7). When the reaction was carried out in BMIm-BF_4_ using anodically generated BF_3_, good yields of product **13** were obtained at room temperature (Table 4, entry 1), while better results were achieved at 60 °C (Table 4, entries 2 and 3). When 4-clorobenzaldehyde was used, the yield was slightly lower (Table 4, entries 4 and 5), but comparable with the literature (entry 7).

In conclusion, we efficiently *in situ* generated BF_3_ via direct anodic oxidation of BMIm-BF_4_ solutions. By simply using electrons as redox reagents, precise control of the amount of formed BF_3_ could be reached and the anolyte could be used directly to carry out organic reactions. This setup was successfully applied to four classically BF_3_-catalyzed transformations, affording similar or improved yields compared with literature results. Moreover, the eco-friendly nature of the developed methodology was demonstrated by the recycling of the IL, which was submitted to up to five subsequent runs without any reactivity loss. We believe that this could be a safer and easier approach to handle this toxic and volatile reagent without storing need and to carry out organic transformations in a sustainable way.

## Experimental Section

### General Infomation

All chemicals were commercial (Fluorochem, Aldrich) and used without further purification. BMIm-BF_4_ (1-butyl-3-methylimidazolium tetrafluoroborate, Iolitec) was kept at 40 °C under vacuum for 3 h before use. ^1^H and ^13^C spectra were recorded at ambient temperature on a Bruker Avance spectrometer (400 MHz) or with a Gemini Varian spectrometer (300 MHz), using the solvent as internal standard. The chemical shifts (δ) are given in ppm relative to TMS. GC–MS analyses have been run on an HP 5892 series II GC, equipped with a 5% phenyl silicone 30m × 0.25 mm × 25 mm capillary column and coupled to an HP 5972 MSD instrument operating at 70 eV. Flash column chromatography was carried out using a Merck 60 kieselgel (230–400 mesh) under pressure. Starting compounds **1**, **2**, **5**, **6**, **7**, **9**, **10**, and **12** were commercially available (Sigma-Aldrich) and used as received.

### General Procedure for Electrochemical BF_3_ Production

All the experiments were carried out in a homemade divided glass cell separated through a porous glass plug; Pt spirals (apparent area 0.8 cm^2^) were used as anode and cathode. Electrolyses were performed at constant current (*I* = 10 mA cm^–2^), at room temperature, under a nitrogen atmosphere, using an Amel Model 552 potentiostat equipped with an Amel Model 731 integrator. 3.0 mL of BMImBF_4_ was put in the anodic compartment, 1.0 mL of BMImBF_4_ in the cathodic one. After a predetermined number of Coulombs (as reported in tables) passed through the electrolysis cell, the current was switched off, the cathodic compartment was removed, and the reagents were added to the anolyte under an inert atmosphere, as specified below. At the end of the reaction, the anolyte was extracted with diethyl ether (3 × 10 mL). The solvent was eliminated from the combined organic phases under reduced pressure, the crude was analyzed by ^1^H NMR, and then the products were purified by flash column chromatography.

When the same anolyte was reused in subsequent electrolyses/experiments, prior to its reuse it, was kept under vacuum for 30 min to eliminate diethyl ether residues.

All products were known, and their spectral data were in accordance with those reported in the literature.

### Friedel–Crafts/Lactonization Reaction

The electrolysis was carried out as previously reported, and after the number of Coulombs reported in Table 1, the current was switched off. Then phenol **1** (0.5 mmol, 1 equiv) and diethyl ketomalonate **2** (87 mg, 0.5 mmol, 1 equiv) were added to the anolyte. The mixture was kept at room temperature under stirring at the temperature and for the time reported in Table 1 and then was extracted with diethyl ether (3 × 10 mL).

#### Ethyl 3-Hydroxy-5-methoxy-2-oxo-2,3-dihydrobenzofuran-3-carboxylate (**3a**)^13^

The product was isolated after flash chromatography on silica gel (light petroleum ether/EtOAc 7:3) as a yellow oil, 100 mg (79%). ^1^H NMR (300 MHz, CDCl3) δ 7.08 (d, *J* = 8.8 Hz, 1H), 6.95 (d, *J* = 8.8 Hz, 1H), 6.84 (s, 1H), 4.44 (s, 1H), 4.16–4.39 (m, 2H), 3.79 (s, 3H), 1.20 (t, *J* = 7.1 Hz, 3H). ^13^C{^1^H} NMR (75 MHz, CDCl3) δ 172.1, 168.5, 157.1, 148.1, 126.0, 117.4, 112.2, 109.3, 76.8, 64.1, 55.9, 13.7. GC–MS, *m*/*z* (%): 253 (M^+·^ +1, 3), 252 (M^+·^, 21), 224 (8), 180 (11), 179 (28), 152 (9), 151 (100), 150 (21), 135 (6), 123 (11), 108 (13), 106 (7), 95 (15), 80 (8), 79 (12), 65 (8), 63 (12), 55 (5), 54 (7), 53 (19), 52 (20), 51 (11), 43 (5), 41 (6).

#### Diethyl 2-Hydroxy-2-(2-hydroxy-5-methoxyphenyl)malonate (**4a**)^13^

The product was isolated after flash chromatography on silica gel (light petroleum ether/EtOAc 7:3) as a white solid, 28 mg (19%). ^1^H NMR (300 MHz, CDCl3) δ 7.14 (s, 1H), 6.97–6.75 (m, 3H), 4.57 (s, 1H), 4.43–4.24 (m, 4H), 3.76 (s, 3H), 1.32 (t, *J* = 7.1 Hz, 6H). ^13^C{^1^H} NMR (75 MHz, CDCl3) δ 169.5, 153.1, 148.8, 122.7, 119.1, 115.8, 113.3, 80.9, 63.5, 55.8, 14.0.

#### Ethyl 3-Hydroxy-2-oxo-2,3-dihydrobenzofuran-3-carboxylate (**3b**)^13^

The product was isolated after flash chromatography on silica gel (light petroleum ether/EtOAc 7:3) as a yellow oil, 98 mg (88%). ^1^H NMR (300 MHz, CDCl3) δ 7.52–7.05 (m, 4H), 4.25 (dtd, *J* = 24.9, 17.7, 7.3 Hz, 2H), 1.17 (t, *J* = 7.1 Hz, 3H). ^13^C{^1^H} NMR (75 MHz, CDCl3) δ 171.9, 168.6, 154.5, 131.9, 125.5, 125.2, 124.3, 111.6, 76.4, 64.2, 13.9. GC–MS, *m*/*z* (%): 222 (M^+·^ +1, 2), 160 (2), 151 (5), 150 (67), 149 (100), 133 (2), 122 (7), 121 (74), 120 (6), 105 (23), 104 (6), 94 (2), 93 (28), 92 (20), 78 (2), 77 (14), 75 (14), 74 (4),72 (2), 66 (11), 65 (57), 64 (19), 63 (20), 61 (5), 55 (2), 53 (12), 51 (16), 49 (6), 44 (10), 43 (8), 40 (5).

#### Ethyl 1-Hydroxy-2-oxo-1,2-dihydronaphtho[2,1-*b*]furan-1-carboxylate (**3c**)^13^

The product was isolated after flash chromatography on silica gel (light petroleum ether/EtOAc 7:3) as a yellow solid, 117 mg (86%). ^1^H NMR (300 MHz, CDCl3) δ 7.97 (d, *J* = 8.9 Hz, 1H), 7.90 (d, *J* = 8.3 Hz, 1H), 7.78 (d, *J* = 8.3 Hz, 1H), 7.57 (ddd, *J* = 8.3, 6.9, 1.2 Hz, 1H), 7.48 (ddd, *J* = 8.2, 6.9, 1.2 Hz, 1H), 7.38 (d, *J* = 8.9 Hz, 1H), 4.62 (s, 1H), 4.24 (ddq, *J* = 55.1, 10.7, 7.1 Hz, 2H), 1.11 (t, *J* = 7.1 Hz, 3H). ^13^C{^1^H} NMR (75 MHz, CDCl3) δ 172.4, 169.1, 153.2, 133.1, 131.2, 129.4, 129.0, 128.8, 125.6, 122.2, 117.5, 111.7, 77.3, 64.4, 13.9. GC–MS, *m*/*z* (%): 272 (M^+·^ +1, 25), 200 (29), 199 (100), 172 (11), 171 (84), 155 (8), 143 (18), 127 (5), 126 (9), 116 (9), 115 (94), 114 (21), 113 (9), 89 (14), 88 (8), 65 (6), 63 (13), 62 (5).

### Povarov Reaction

#### Imine Synthesis

Amine **5** (0.5 mmol, 1 equiv) and aldehyde **6** (0.5 mmol, 1 equiv) were added to 0.5 mL of BMIm-BF_4_ and kept under stirring at room temperature for 1 h. Then the mixture was extracted with diethyl ether (3 × 3 mL). The solvent was eliminated from the combined organic phases under reduced pressure, and the imine was used without purification (after ^1^H NMR control spectrum) in the Povarov reaction.

The electrolysis was carried out as previously reported, and after the number of Coulombs reported in Table 2, the current was switched off. Then imine (0.5 mmol, 1 equiv) and 3,4-dihydro-2*H*-pyrane **7** (1–4 equiv, amount as in Table 2) were added to the anolyte. The mixture was kept at room temperature under stirring and an inert atmosphere for 3 h, then extracted with diethyl ether.

##### 9-Methyl-5-phenyl-3,4,4a,5,6,10b-hexahydro-2*H*-pyrano[3,2-*c*]quinoline (**8a**)^20^

The *cis* product was isolated after crystallization from ethanol, the *trans* product after flash chromatography on silica gel (light petroleum ether/EtOAc 9:1) of the mother liquor.

*Cis*, white solid, 95 mg (68%): ^1^H NMR (400 MHz, CDCl3) δ 7.45–7.34 (m, 4H), 7.34–7.28 (m, 1H), 7.26 (s, 1H), 6.93 (dd, *J* = 8.1, 2.0 Hz, 1H), 6.54 (d, *J* = 8.0 Hz, 1H), 5.32 (d, *J* = 5.5 Hz, 1H), 4.66 (d, *J* = 2.4 Hz, 1H), 3.78 (bs, 1H), 3.63–3.57 (m, 1H), 3.45 (td, *J* = 11.5, 2.5 Hz, 1H), 2.29 (s, 3H), 2.21–2.12 (m, 1H), 1.63–1.40 (m, 3H), 1.36–1.27 (m, 1H). ^13^C{^1^H} NMR (101 MHz, CDCl3) δ 142.9, 141.4, 128.9, 128.4, 127.9, 127.6, 126.9, 120.0, 114.7, 73.0, 60.8, 59.6, 39.2, 25.6, 20.8, 18.1. GC–MS, *m*/*z* (%): 280 (M^+·^ +1, 21), 279 (M^+·^, 100), 264 (4), 248 (16) 239 (19), 220 (81), 208 (43), 144 (31).

*Trans*, light yellow oil, 39 mg (28%): ^1^H NMR (400 MHz, CDCl3) δ 7.38 (ddd, *J* = 21.8, 16.3, 7.3 Hz, 5H), 7.06 (s, 1H), 6.92 (d, *J* = 8.1 Hz, 1H), 6.47 (d, *J* = 8.1 Hz, 1H), 4.70 (d, *J* = 10.8 Hz, 1H), 4.37 (d, *J* = 2.5 Hz, 1H), 4.15–4.08 (m, 1H), 3.99 (bs, 1H), 3.73 (td, *J* = 11.6, 2.4 Hz, 1H), 2.25 (s, 3H), 2.13–2.04 (m, 1H), 1.85 (dddd, *J* = 17.5, 13.6, 9.0, 4.6 Hz, 1H), 1.65 (tt, *J* = 13.3, 4.6 Hz, 1H), 1.47 (d, *J* = 13.6 Hz, 1H), 1.33 (d, *J* = 13.3 Hz, 1H). ^13^C{^1^H} NMR (101 MHz, CDCl3) δ 142.5, 142.5, 131.1, 130.1, 128.6, 127.9, 126.7, 120.7, 114.3, 74.6, 68.7, 54.9, 39.1, 24.2, 22.0, 20.4. GC–MS, *m*/*z* (%): 280 (M^+·^ +1, 14), 279 (M^+·^, 70), 248 (9) 234 (13), 220 (100), 208 (22), 144 (24).

##### 9-Methoxy-5-phenyl-3,4,4a,5,6,10b-hexahydro-2*H*-pyrano[3,2-*c*]quinoline (**8b**)^16^

The *cis* product was isolated after crystallization from ethanol, the *trans* product after chromatography on silica gel (light petroleum ether/EtOAc 9:1) of the mother liquor.

*Cis*, white solid, 121 mg (82%): ^1^H NMR (400 MHz, CDCl3) δ 7.44 (d, *J* = 7.0 Hz, 2H), 7.41–7.35 (m, 2H), 7.34–7.29 (m, 1H), 7.07–7.04 (m, 1H), 6.74 (ddd, *J* = 8.6, 2.9, 0.7 Hz, 1H), 6.58 (d, *J* = 8.6 Hz, 1H), 5.32 (d, *J* = 5.6 Hz, 1H), 4.63 (d, *J* = 2.2 Hz, 1H), 3.79 (s, 3H), 3.69 (bs, 1H), 3.64–3.58 (m, 1H), 3.45 (td, *J* = 11.4, 2.5 Hz, 1H), 2.21–2.13 (m, 1H), 1.64–1.41 (m, 3H), 1.38–1.29 (m, 1H). ^13^C{^1^H} NMR (101 MHz, CDCl3) δ 153.0, 141.4, 139.2, 128.4, 127.5, 126.9, 121.2, 115.8, 115.2, 112.0, 73.0, 61.0, 59.7, 56.0, 39.2, 25.5, 18.0. GC–MS, *m*/*z* (%): 296.1 (M^+·^ +1, 24), 295.1 (M^+·^, 100), 236 (43), 236 (43), 224 (33), 159.9 (19), 90.9 (12).

*Trans*, orange oil, 10 mg (7%): ^1^H NMR (400 MHz, CDCl3) δ 7.45–7.28 (m, 5H), 6.82 (d, *J* = 2.9 Hz, 1H), 6.74 (dd, *J* = 8.7, 2.9 Hz, 1H), 6.51 (d, *J* = 8.7 Hz, 1H), 4.67 (d, *J* = 10.7 Hz, 1H), 4.38 (d, *J* = 2.8 Hz, 1H), 4.13–4.06 (m, 1H), 3.77 (s, 3H), 3.72 (td, *J* = 11.5, 2.6 Hz, 1H), 2.15–2.07 (m, 1H), 1.65–1.41 (m, 5H). ^13^C{^1^H} NMR (101 MHz, CDCl3) δ 152.3, 142.5, 135.3, 131.0, 128.8, 128.0, 125.2, 117.0, 115.8, 115.0, 74.1, 68.7, 56.1, 55.4, 39.1, 24.3, 22.2. GC–MS, *m*/*z* (%): 296.1 (M^+·^ +1, 20), 295 (M^+·^, 100), 277.1 (18), 237 (13), 236 (68), 224 (20), 193 (11), 160 (18), 146.9 (20), 117 (10), 115 (10), 91 (14).

##### 5-(4-Methoxyphenyl)-9-methyl-3,4,4a,5,6,10b-hexahydro-2*H*-pyrano[3,2-*c*]quinoline (**8c**)^21^

The *cis* product was isolated after crystallization from ethanol, the *trans* product after chromatography on silica gel (light petroleum ether/EtOAc 9:1) of the mother liquor.

*Cis*, white solid, 70 mg (45%): ^1^H NMR (400 MHz, CDCl3) δ 7.36–7.31 (m, 2H), 7.26–7.24 (m, 1H), 6.94–6.89 (m, 3H), 6.53 (d, *J* = 8.0 Hz, 1H), 5.29 (d, *J* = 5.6 Hz, 1H), 4.60 (d, *J* = 2.4 Hz, 1H), 3.83 (s, 3H), 3.75 (bs, 1H), 3.64–3.56 (m, 1H), 3.44 (td, *J* = 11.4, 2.5 Hz, 1H), 2.28 (s, 3H), 2.17–2.06 (m, 1H), 1.57–1.33 (m, 4H). ^13^C{^1^H} NMR (101 MHz, CDCl3) δ 159.0, 143.0, 133.4, 128.8, 128.0, 127.9, 127.6, 120.0, 114.6, 113.8, 73.0, 60.9, 59.1, 55.4, 39.4, 25.6, 20.8, 18.1. GC–MS, *m*/*z* (%): 310.1 (M^+·^+1, 22), 309.1 (M^+·^, 100), 308.1 (10), 276 (17), 264.1 (11), 251 (14), 250 (71), 239 (14), 238 (71), 145 (19), 144 (28), 121 (35).

*Trans*, orange oil, 37 mg (24%): ^1^H NMR (400 MHz, CDCl3) δ 7.34 (d, *J* = 8.6 Hz, 2H), 7.04 (d, *J* = 1.5 Hz, 1H), 6.90 (d, *J* = 8.7 Hz, 3H), 6.45 (d, *J* = 8.1 Hz, 1H), 4.65 (d, *J* = 10.8 Hz, 1H), 4.36 (d, *J* = 2.7 Hz, 1H), 4.14–4.07 (m, 1H), 3.82 (s, 3H), 3.72 (td, *J* = 11.7, 2.4 Hz, 1H), 2.23 (s, 3H), 2.06 (d, *J* = 4.6 Hz, 1H), 1.65–1.55 (m, 5H). ^13^C{^1^H} NMR (101 MHz, CDCl3) δ 159.2, 135.2, 131.1, 130.1, 128.9, 125.0, 114.0, 74.7, 68.8, 55.3, 54.3, 29.7, 24.2, 21.9, 20.4. GC–MS, *m*/*z* (%): 310 (M^+·^ +1, 21), 309.1 (M^+·^, 83), 280.9 (12), 278 (18), 251.1 (12), 250 (57), 238.9 (24), 238.1 (11), 206.9 (29), 159.9 (0), 120.9 (0).

### Friedel–Craft Benzylation of Anisole with Benzyl Alcohol

The electrolysis was carried out as previously reported, and after the number of Coulombs reported in Table 3, the current was switched off. Then anisole **10** (2–4 equiv, amount as in Table 3) and benzyl alcohol **9** (54 mg, 0.5 mmol, 1 equiv) were added to the anolyte. The mixture was kept at room temperature under stirring and an inert atmosphere for 4 h, then extracted with diethyl ether.

#### 1-Benzyl-4-methoxybenzene (*p*-**11**)^22^

The product was isolated after flash chromatography on silica gel (light petroleum ether/EtOAc 9:1), deliquescent light yellow solid, 46 mg (46%). ^1^H NMR (400 MHz, CDCl3) δ 7.32–7.25 (m, 2H), 7.19 (t, *J* = 7.6 Hz, 3H), 7.11 (d, *J* = 8.4 Hz, 2H), 6.84 (d, *J* = 8.4 Hz, 2H), 3.94 (s, 2H), 3.79 (s, 3H). ^13^C{^1^H} NMR (101 MHz, CDCl3) δ 158.0, 141.6, 133.2, 129.9, 128.8, 128.4, 126.0, 113.9, 55.3, 41.0. GC–MS, *m*/*z* (%): 199 (M^+·^ +1, 15), 198 (M^+·^, 100), 183 (15) 167 (35), 165 (24), 153 (17), 121 (23), 91 (8).

#### 1-Benzyl-2-methoxybenzene (*o*-**11**)^22^

The product was isolated after flash chromatography on silica gel (light petroleum ether/EtOAc 9:1), deliquescent light yellow solid, 34 mg (34%). ^1^H NMR (400 MHz, CDCl3) δ 7.31–7.16 (m, 9H), 7.08 (d, *J* = 6.2 Hz, 1H), 6.89 (m, 2H), 3.99 (s, 2H), 3.83 (s, 3H). ppm. ^13^C{^1^H} NMR (101 MHz, CDCl3) δ 157.3, 141.0, 130.3, 129.7, 129.0, 128.2, 127.4, 125.8, 120.5, 110.4, 55.4, 35.9. GC–MS, *m*/*z* (%): 199 (M^+·^ +1, 16), 198 (M^+·^, 100), 183 (36) 167 (37), 165 (52), 152 (15), 121 (7), 91 (22).

### Multicomponent Reaction to Tetrahydro-11*H*-benzo[*a*]xanthen-11-ones

The electrolysis was carried out as previously reported, and after the number of Coulombs reported in Table 4, the current was switched off. Then benzaldehyde **6** (0.5 mmol, 1 equiv), 2-naphthol **1c** (72 mg, 0.5 mmol, 1 equiv), and dimedone **12** (70 mg, 0.5 mmol, 1 equiv) were added to the anolyte. The mixture was kept at room temperature under stirring and an inert atmosphere for 3 h, (or 60 °C using an oil bath for 1 or 2 h), then extracted with diethyl ether. The products were crystallized from ethanol.

#### 9,9-Dimethyl-12-phenyl-8,9,10,12-tetrahydro-11*H*-benzo[*a*]xanthen-11-one (**13a**)^19^

White solid, 154 mg (87%), ^1^H NMR (400 MHz, CDCl3) δ 8.00 (d, *J* = 8.5 Hz, 1H), 7.80–7.74 (m, 2H), 7.43 (ddd, *J* = 8.4, 6.9, 1.4 Hz, 1H), 7.40–7.31 (m, 4H), 7.21–7.15 (m, 2H), 7.09–7.03 (m, 1H), 5.72 (s, 1H), 2.57 (s, 2H), 2.35–2.21 (m, 2H), 1.12 (s, 3H), 0.97 (s, 3H). ^13^C{^1^H} NMR (101 MHz, CDCl_3_) δ 197.0, 164.0, 147.9, 144.9, 131.6, 131.5, 128.9, 128.5, 128.5, 128.3, 127.1, 126.3, 125.0, 123.8, 117.8, 117.2, 114.4, 51.0, 41.5, 34.8, 32.4, 29.4, 27.3. GC–MS, *m*/*z* (%): 354.1 (M^+·^, 33), 278.1 (21), 277.1 (100), 221 (10).

#### 12-(4-Chlorophenyl)-9,9-dimethyl-8,9,10,12-tetrahydro-11*H*-benzo[*a*]xanthen-11-one (**13b**)^19^

White solid, 148 mg (76%), ^1^H NMR (400 MHz, CDCl3) δ 7.92 (d, *J* = 8.0 Hz, 1H), 7.81–7.75 (m, 2H), 7.44 (ddd, *J* = 8.4, 6.9, 1.5 Hz, 1H), 7.39 (ddd, *J* = 8.0, 6.9, 1.3 Hz, 1H), 7.34–7.29 (m, 2H), 7.18–7.13 (m, 2H), 5.71 (s, 1H), 2.56 (s, 2H), 2.35–2.22 (m, 2H), 1.12 (s, 3H), 0.97 (s, 3H). ^13^C{^1^H} NMR (101 MHz, CDCl_3_) δ 196.9, 164.1, 147.8, 143.3, 132.0, 131.6, 131.3, 129.9, 129.2, 128.6, 128.5, 127.2, 125.1, 123.5, 117.1, 113.9, 50.9, 41.4, 34.3, 32.3, 29.4, 27.2 ppm. GC–MS, *m*/*z* (%): 388.1 (M^+·^, 26), 278.1 (21), 277.1 (100), 221 (10).

## Supporting Information Available

Copies of ^1^H and ^13^C NMR of products (PDF)
