# Supplementary material for: In Situ Anodically Oxidized BMIm-BF4: A Safe and Recyclable BF3 Source
Source: J Org Chem. 2021 Jul 2;86(22):16151–7. doi: 10.1021/acs.joc.1c00932 (PMC8609525; doi:10.1021/acs.joc.1c00932)

Supplementary Information

In Situ Anodically Oxidized BMIm-BF_4: a Safe and Recyclable BF_3 Source

Martina Bortolami,[a] Leonardo Mattiello,[a] Vincenzo Scarano,[a] Fabrizio Vetica,[b] Marta Feroci*[a]

[a]*Department of Basic and Applied Sciences for Engineering (SBAI), Sapienza University of Rome, via Castro Laurenziano, 7, 00161 Rome (Italy);marta.feroci@uniroma1.it*
[b]*Department of Chemistry, Sapienza University of Rome, piazzale Aldo Moro, 5, 00185 Rome (Italy)*

Table of Contents

Cyclic Voltammetry of BMIm-BF_4 S2

^{1}H and ^{13}C{^{1}H} NMR spectra in $CDCl_3$:

Ethyl 3-hydroxy-5-methoxy-2-oxo-2,3-dihydrobenzofuran-3-carboxylate (**3a**) S3

Diethyl 2-hydroxy-2-(2-hydroxy-5-methoxyphenyl)malonate (**4a**) S4

Ethyl 3-hydroxy-2-oxo-2,3-dihydrobenzofuran-3-carboxylate (**3b**) S5

Ethyl 1-hydroxy-2-oxo-1,2-dihydronaphtho[2,1-*b*]furan-1-carboxylate (**3c**) S6

cis-9-methyl-5-phenyl-3,4,4a,5,6,10b-hexahydro-2*H*-pyrano[3,2-*c*]quinoline (**8a**) S7

trans-9-methyl-5-phenyl-3,4,4a,5,6,10b-hexahydro-2*H*-pyrano[3,2-*c*]quinoline (**8a**) S8

cis-9-methoxy-5-phenyl-3,4,4a,5,6,10b-hexahydro-2*H*-pyrano[3,2-*c*]quinoline (**8b**) S9

trans-9-methoxy-5-phenyl-3,4,4a,5,6,10b-hexahydro-2*H*-pyrano[3,2-*c*]quinoline (**8b**) S10

cis-5-(4-methoxyphenyl)-9-methyl-3,4,4a,5,6,10b-hexahydro-2*H*-pyrano[3,2-*c*]quinoline (**8c**) S11

trans-5-(4-methoxyphenyl)-9-methyl-3,4,4a,5,6,10b-hexahydro-2*H*-pyrano[3,2-*c*]quinoline (**8c**) S12

1-benzyl-4-methoxybenzene (***p*-11**) S13

1-benzyl-2-methoxybenzene (***o*-11**) S14

9,9-dimethyl-12-phenyl-8,9,10,12-tetrahydro-11*H*-benzo[*a*]xanthen-11-one (**13a**) S15

12-(4-chlorophenyl)-9,9-dimethyl-8,9,10,12-tetrahydro-11*H*-benzo[*a*]xanthen-11-one (**13b**) S16

Cyclic Voltammetry of neat BMIm-BF_4 on Platinum or Glassy Carbon or Gold electrode.

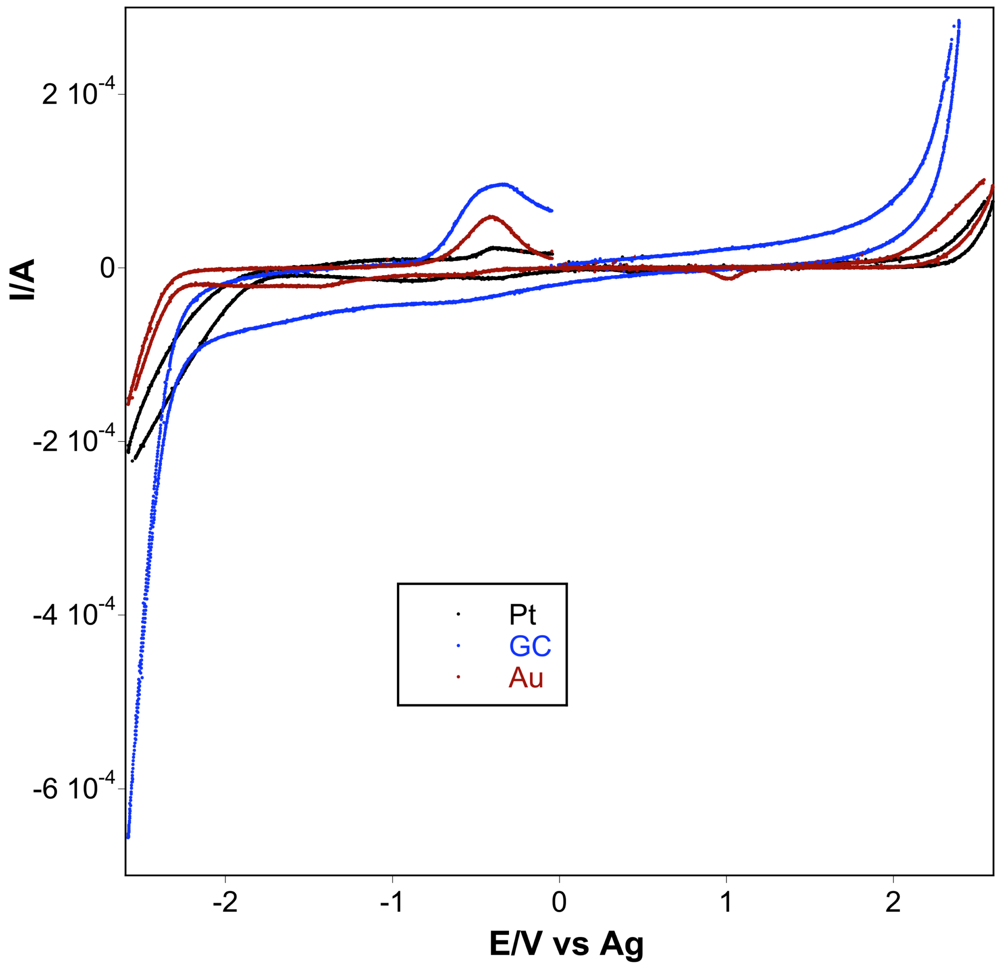

Cyclic voltammetry of neat BMIm-BF_4. Pt (492/PT/1) or GC (492/GC/3) or Au (492/AU/1) Amel working microelectrode, Ag quasi-reference electrode, room temperature, nitrogen atmosphere. Scan rate: 200 mV s^{-1}. Potential scan: 0 to + 2.6 to – 2.6 to 0 V.

Instrument: Amel 552 potentiostat equipped with an Amel 566 function generator and an Amel 563 multipurpose unit in a three-electrode cell; Amel 863 recorder; acquisition software: CorrWare for windows version 2.8d1 Scribner, elaboration software: CorrView for windows version 2.8d1 Scribner.

^{1}H and $^{13}C\{^{1}H\}$ NMR spectra in $CDCl_3$

Ethyl 3-hydroxy-5-methoxy-2-oxo-2,3-dihydrobenzofuran-3-carboxylate (3a)

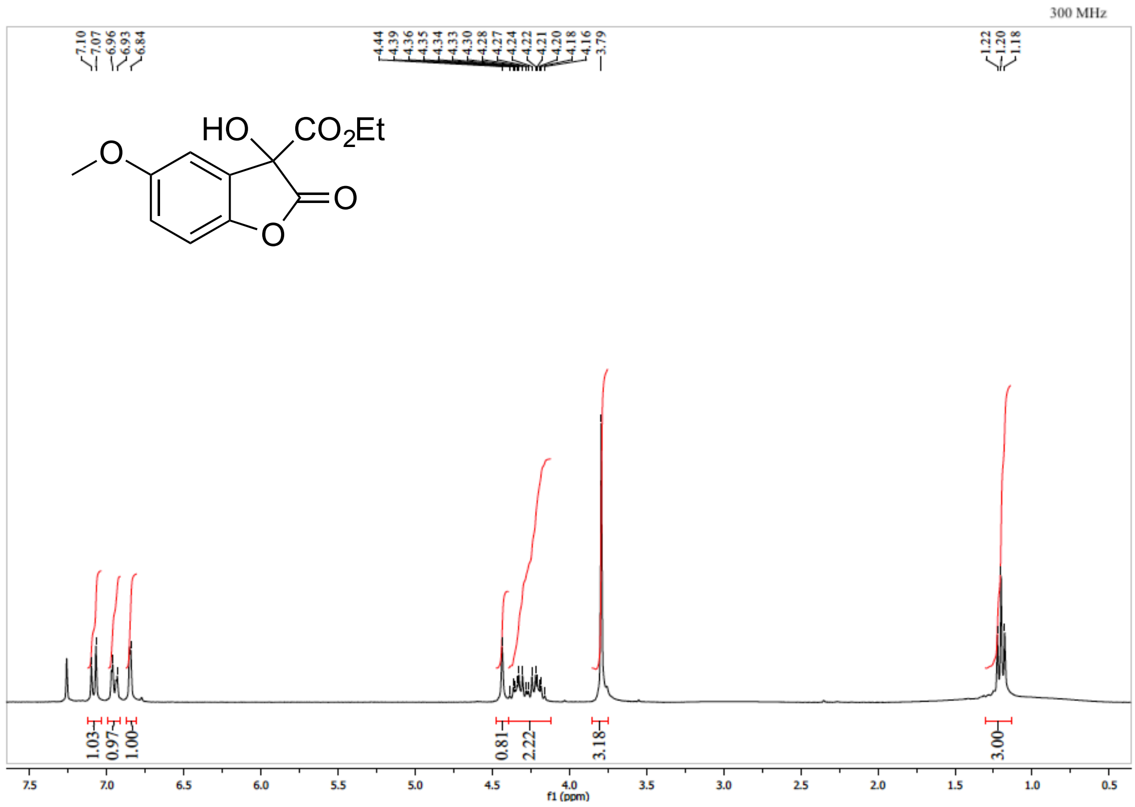

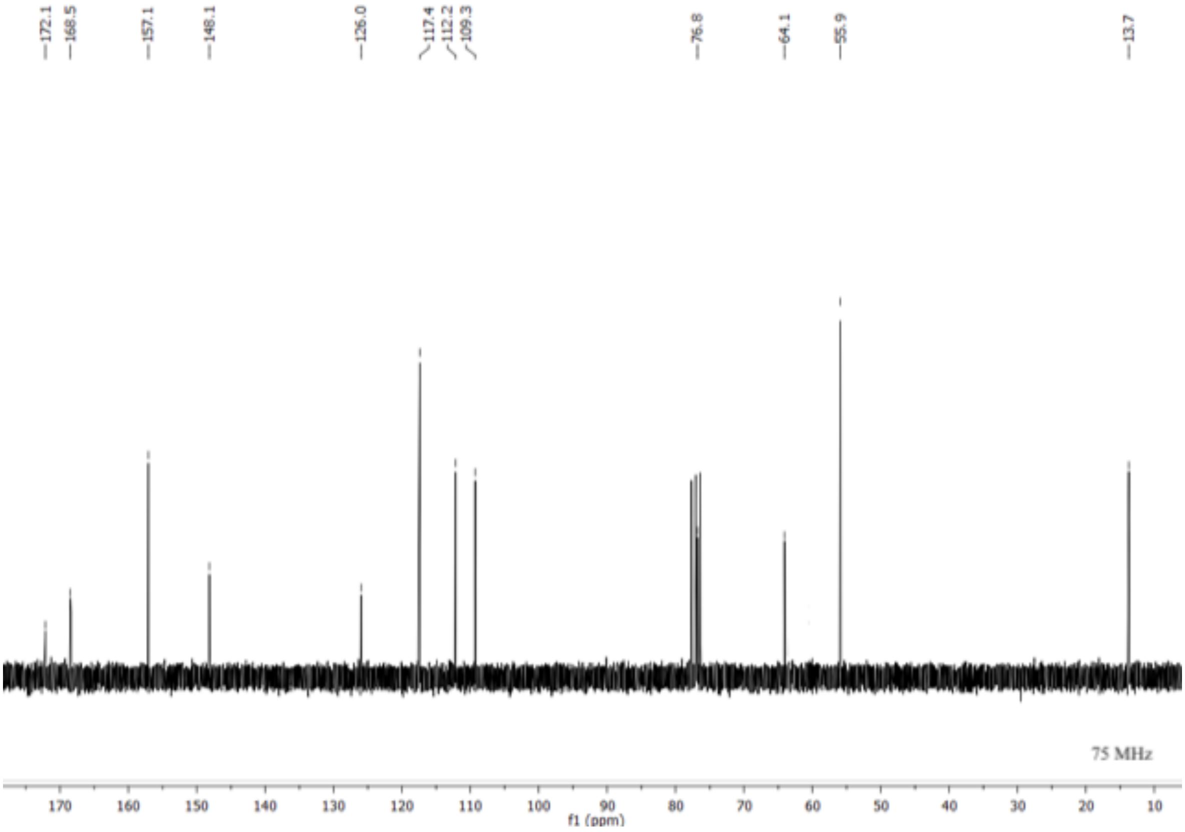

^{1}H and ^{13}C{^{1}H} NMR spectra in $CDCl_3$

Diethyl 2-hydroxy-2-(2-hydroxy-5-methoxyphenyl)malonate (4a)

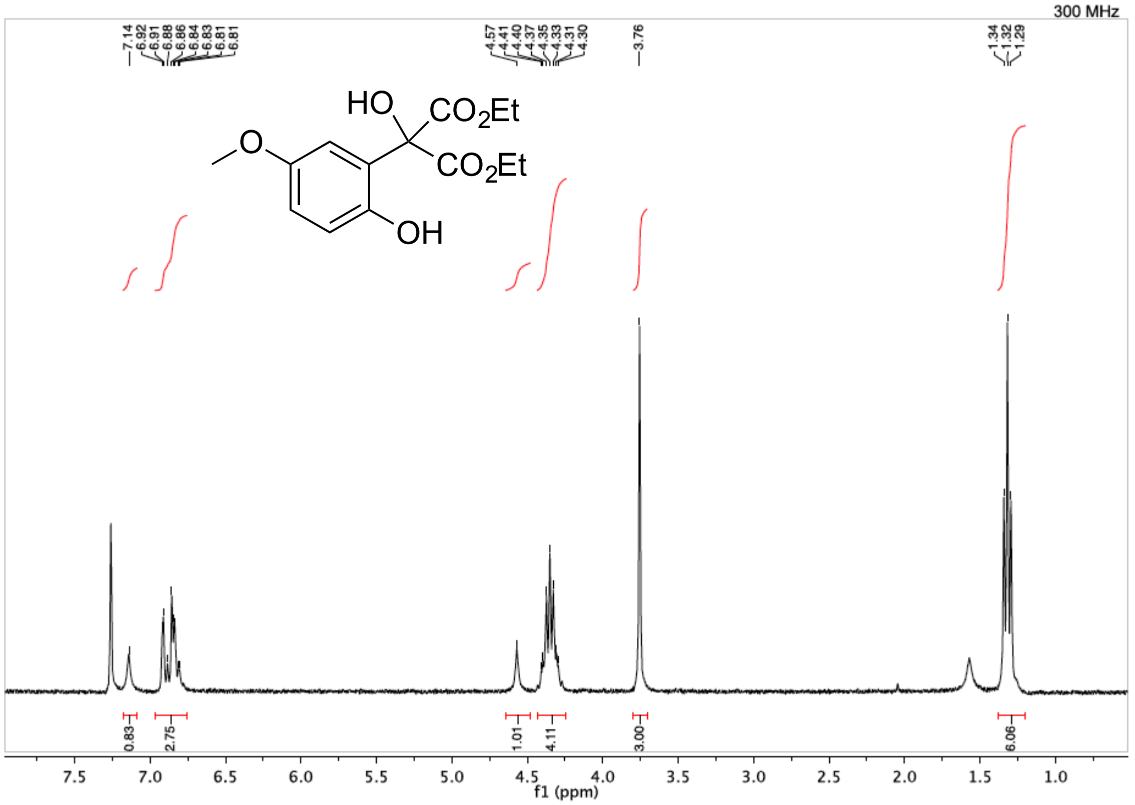

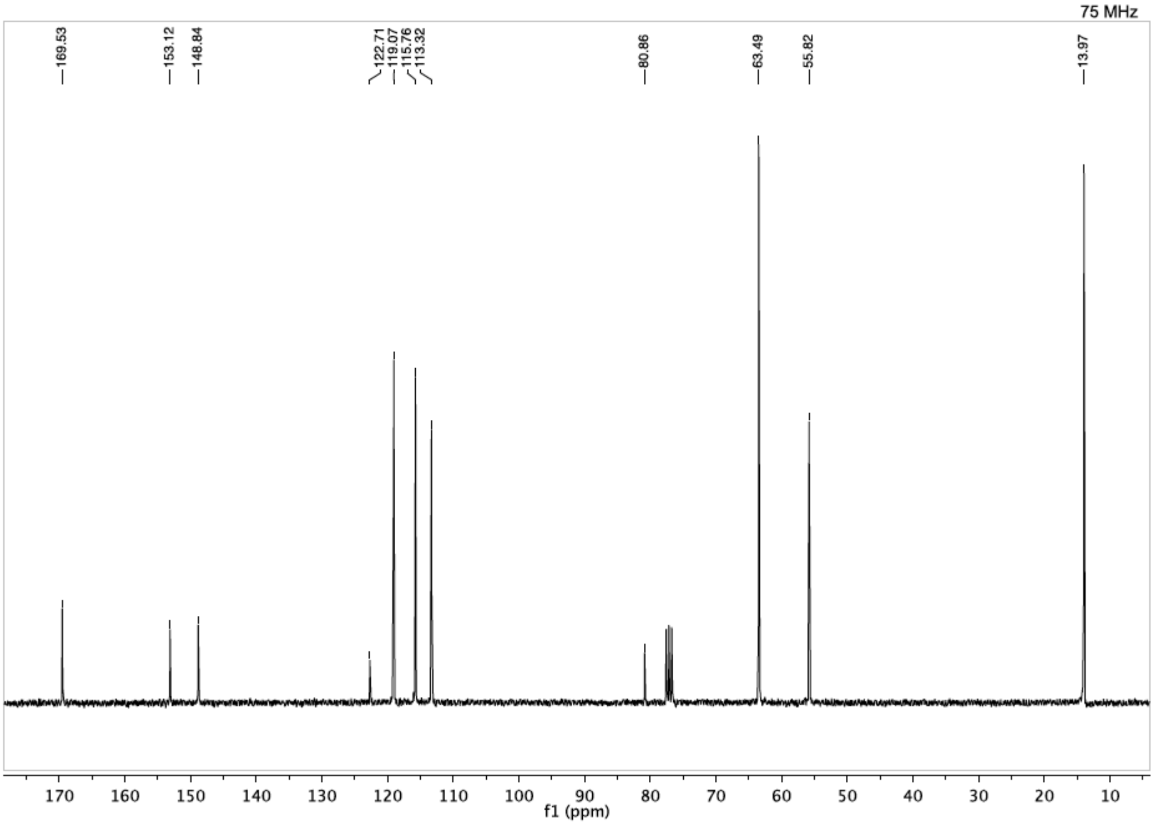

^{1}H and ^{13}C{^{1}H} NMR spectra in $CDCl_3$

Ethyl 3-hydroxy-2-oxo-2,3-dihydrobenzofuran-3-carboxylate (3b)

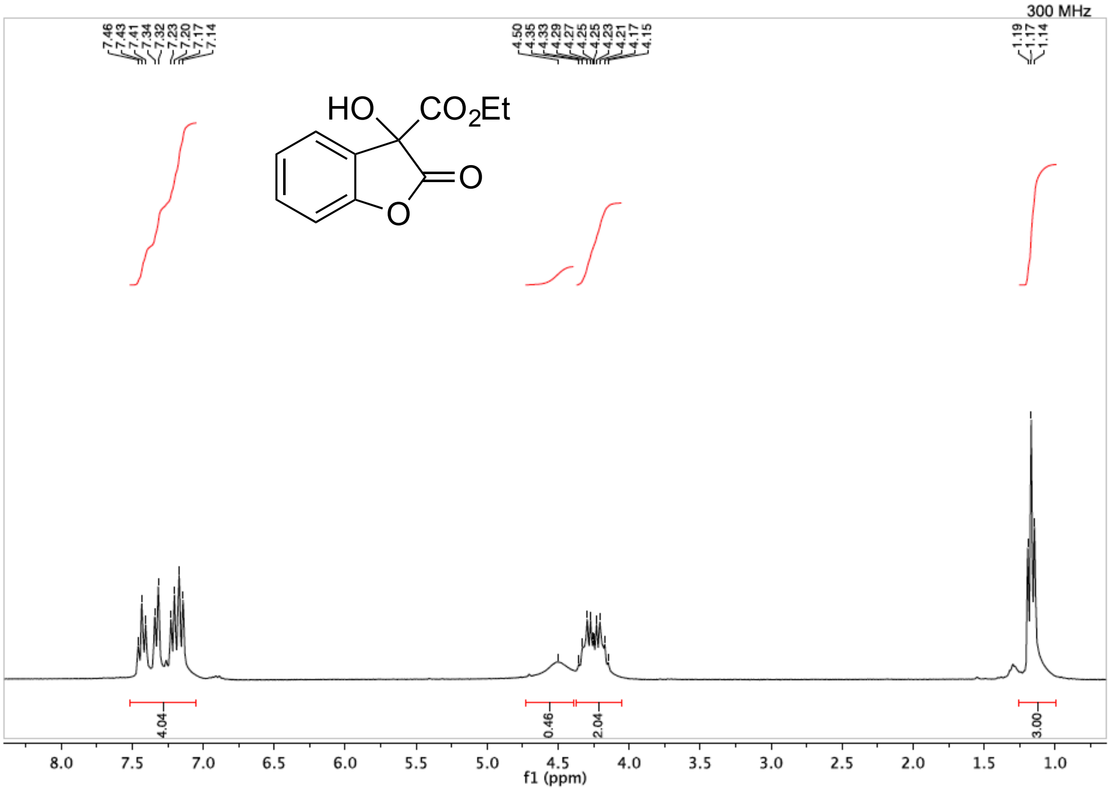

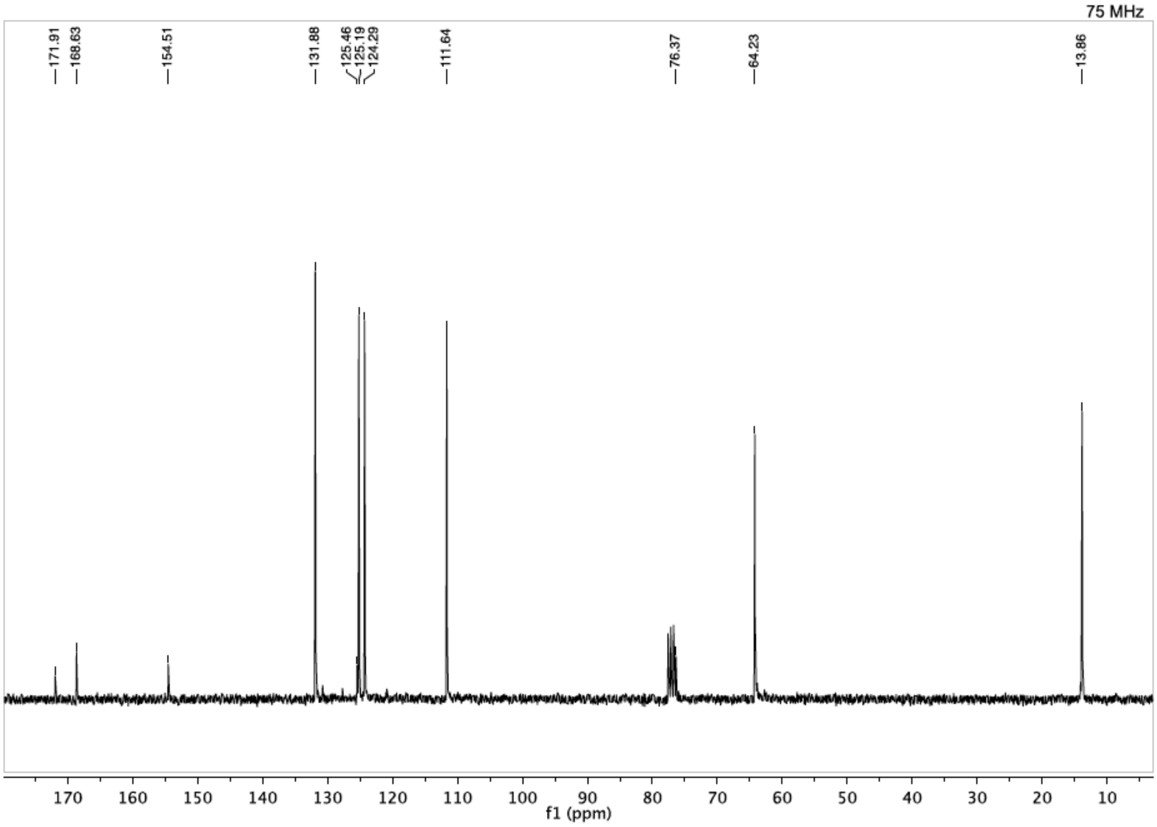

^{1}H and $^{13}C\{^{1}H\}$ NMR spectra in $CDCl_3$

Ethyl 1-hydroxy-2-oxo-1,2-dihydronaphtho[2,1-*b*]furan-1-carboxylate (3c)

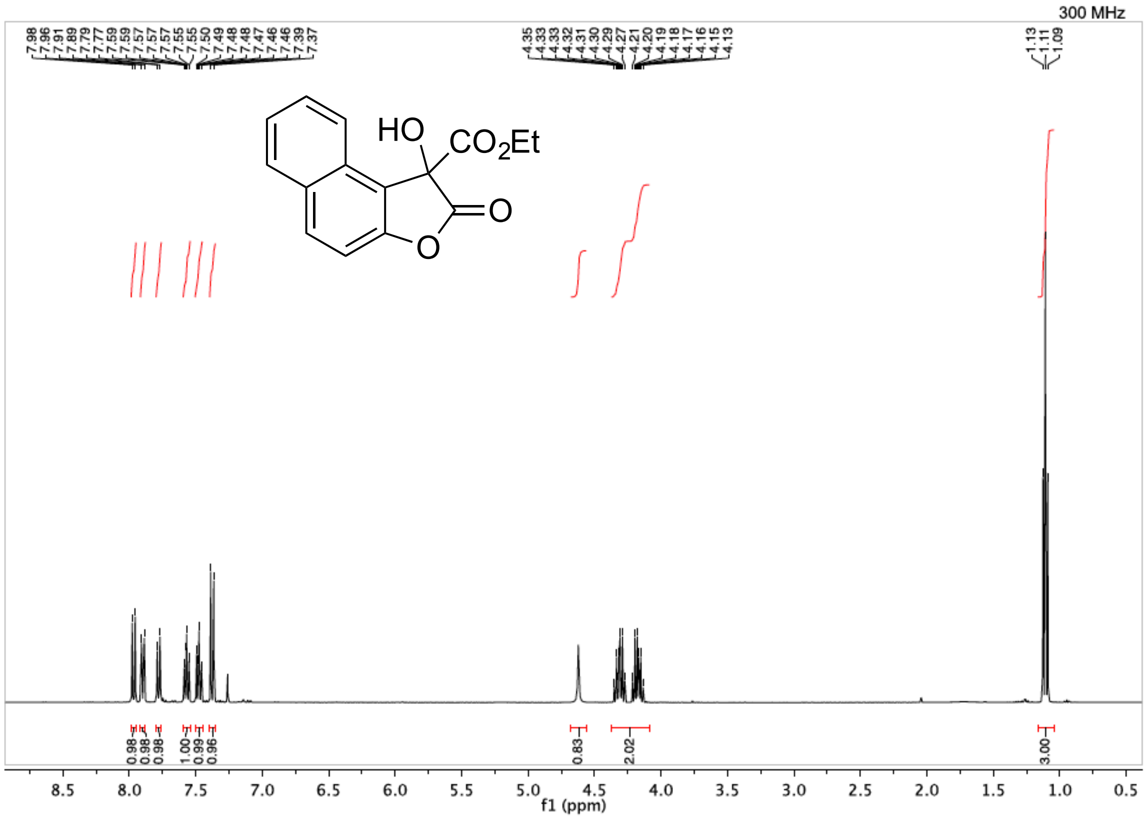

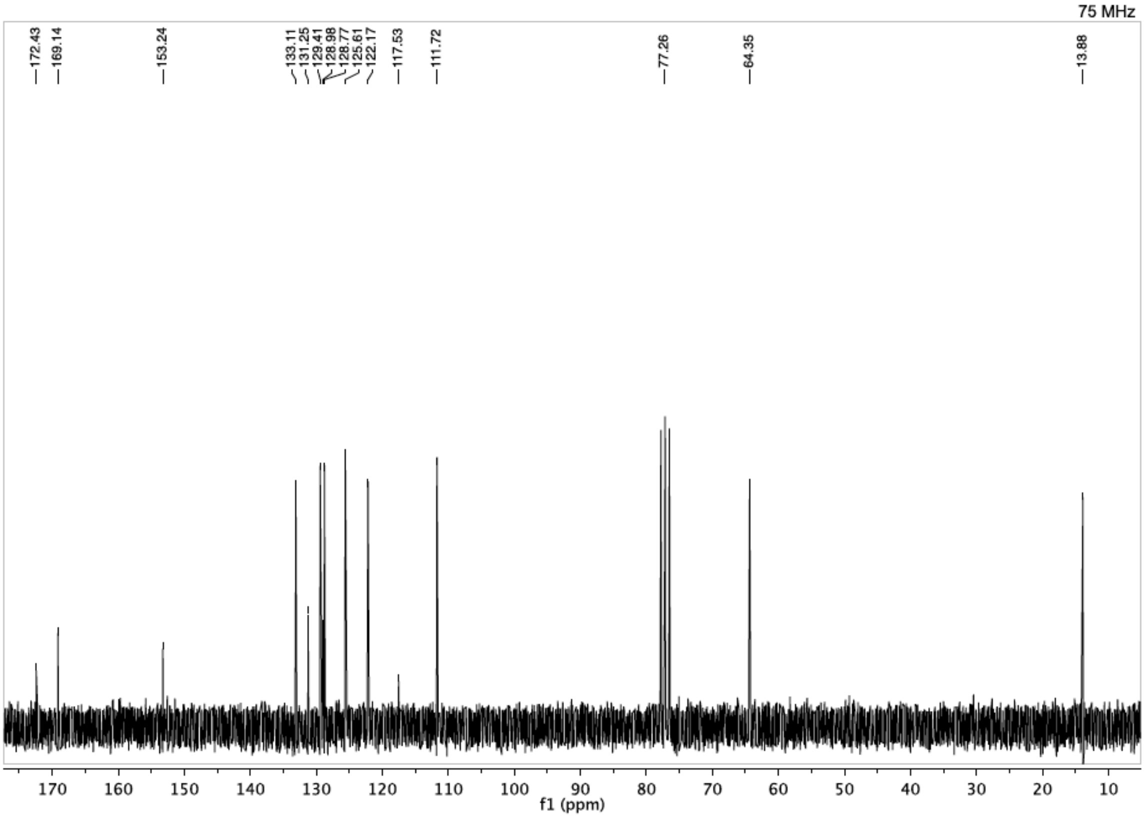

^{1}H and $^{13}C\{^{1}H\}$ NMR spectra in $CDCl_3$

***cis*-9-methyl-5-phenyl-3,4,4a,5,6,10b-hexahydro-2*H*-pyrano[3,2-*c*]quinoline (8a)**

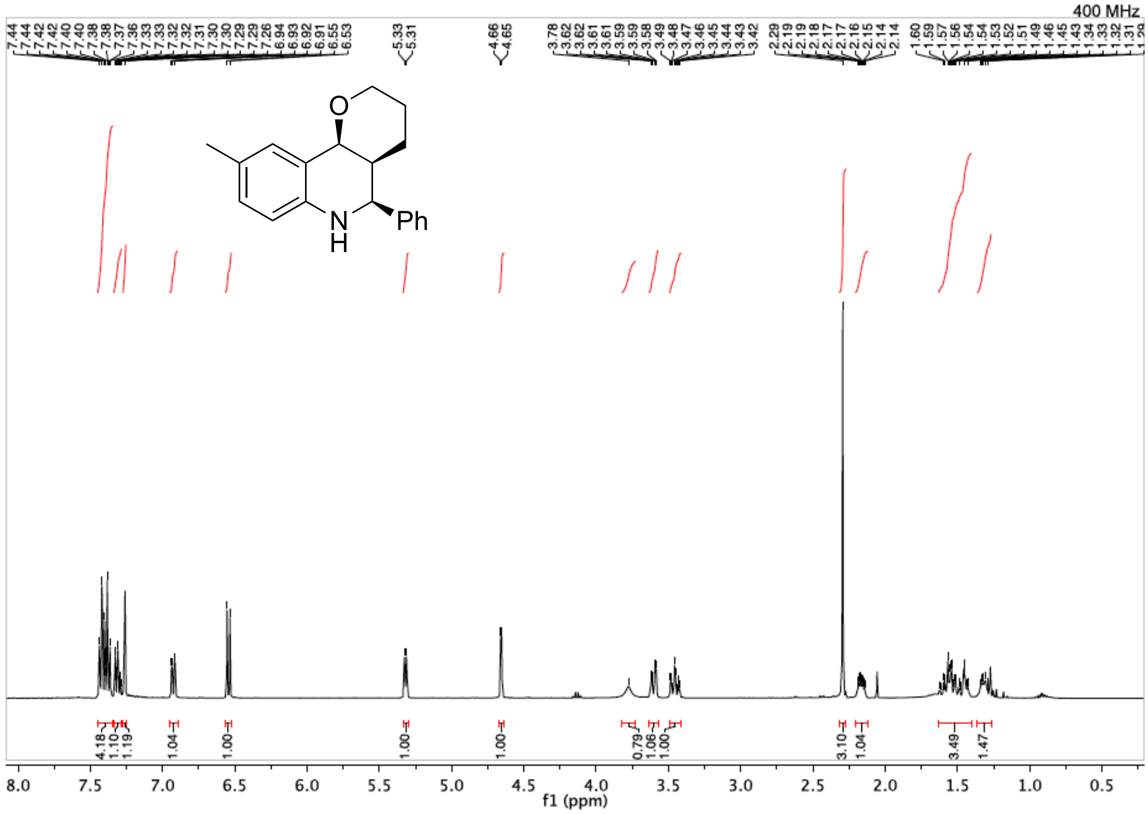

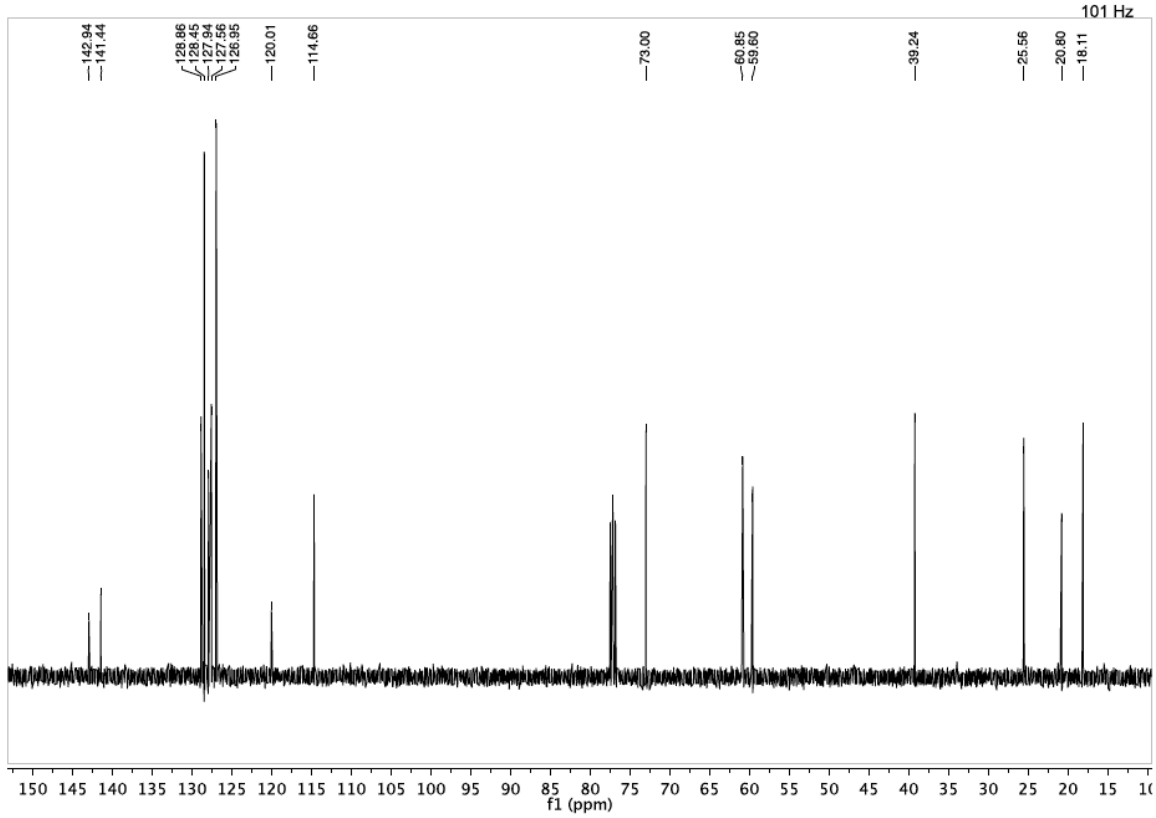

^{1}H and ^{13}C{^{1}H} NMR spectra in $CDCl_3$

***trans*-9-methyl-5-phenyl-3,4,4a,5,6,10b-hexahydro-2*H*-pyrano[3,2-*c*]quinoline (8a)**

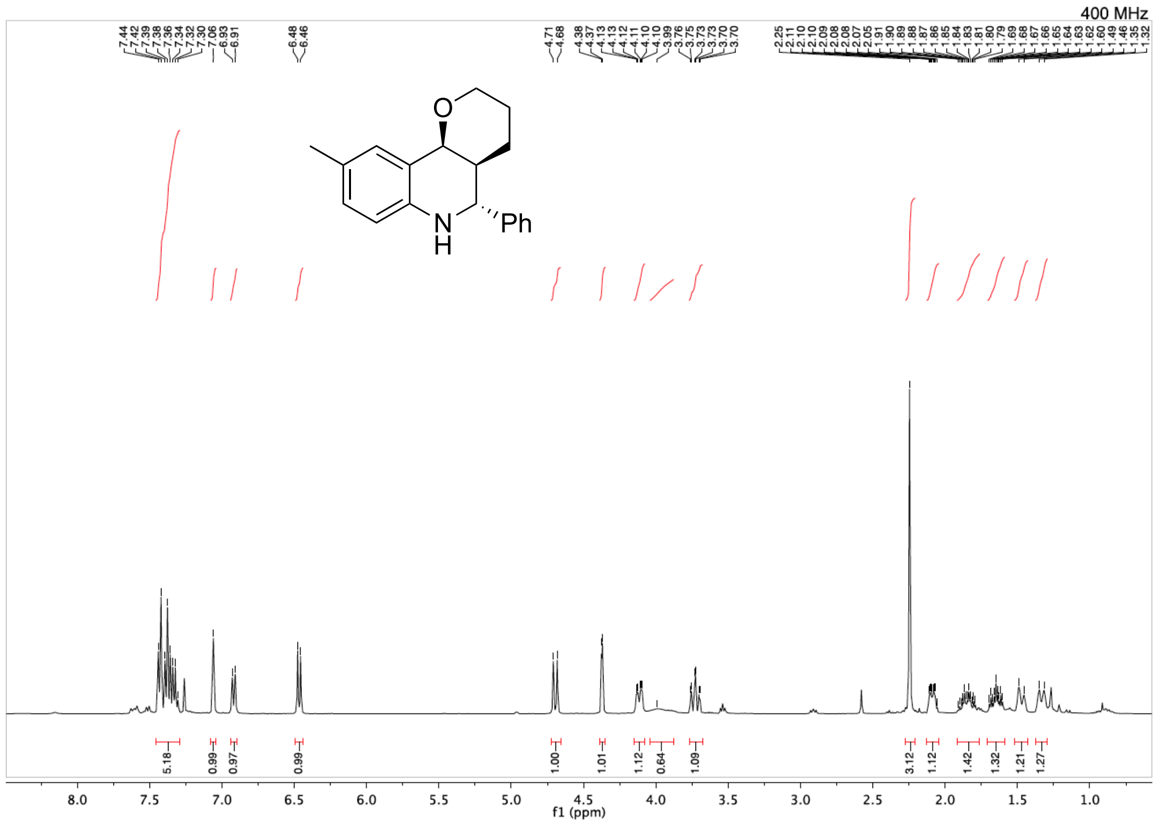

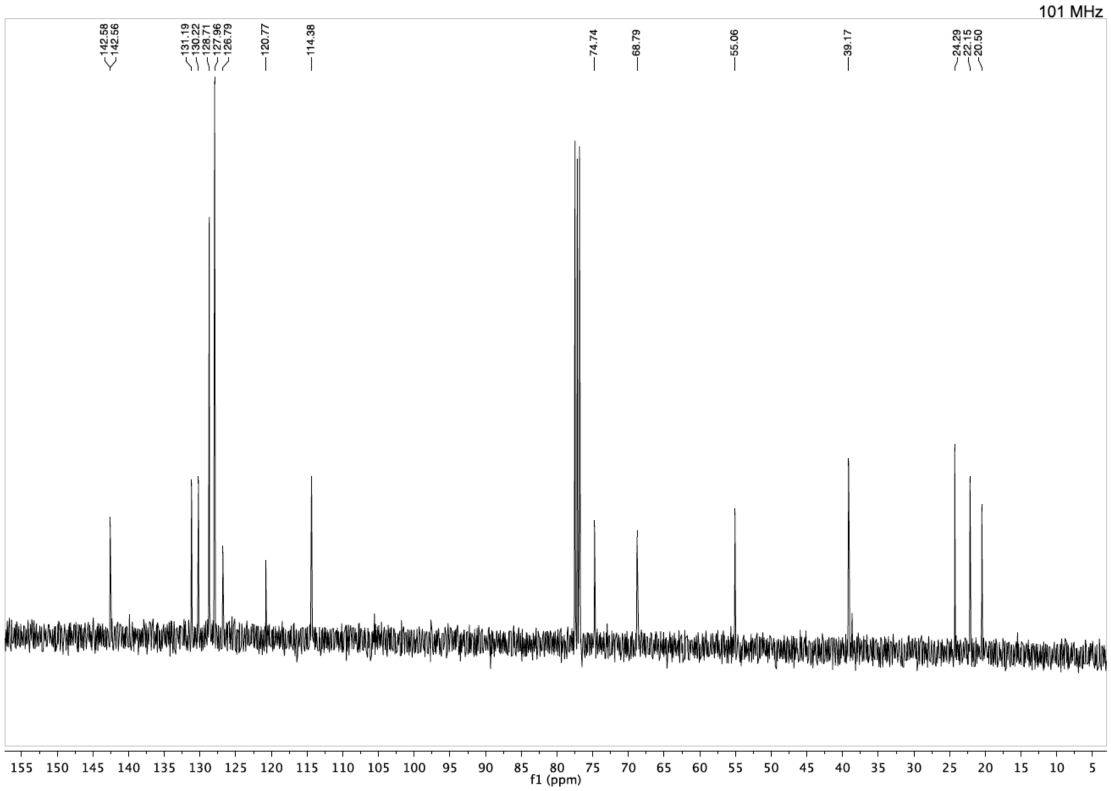

^{1}H and ^{13}C{^{1}H} NMR spectra in $CDCl_3$

cis-9-methoxy-5-phenyl-3,4,4a,5,6,10b-hexahydro-2*H*-pyrano[3,2-*c*]quinoline (8b)

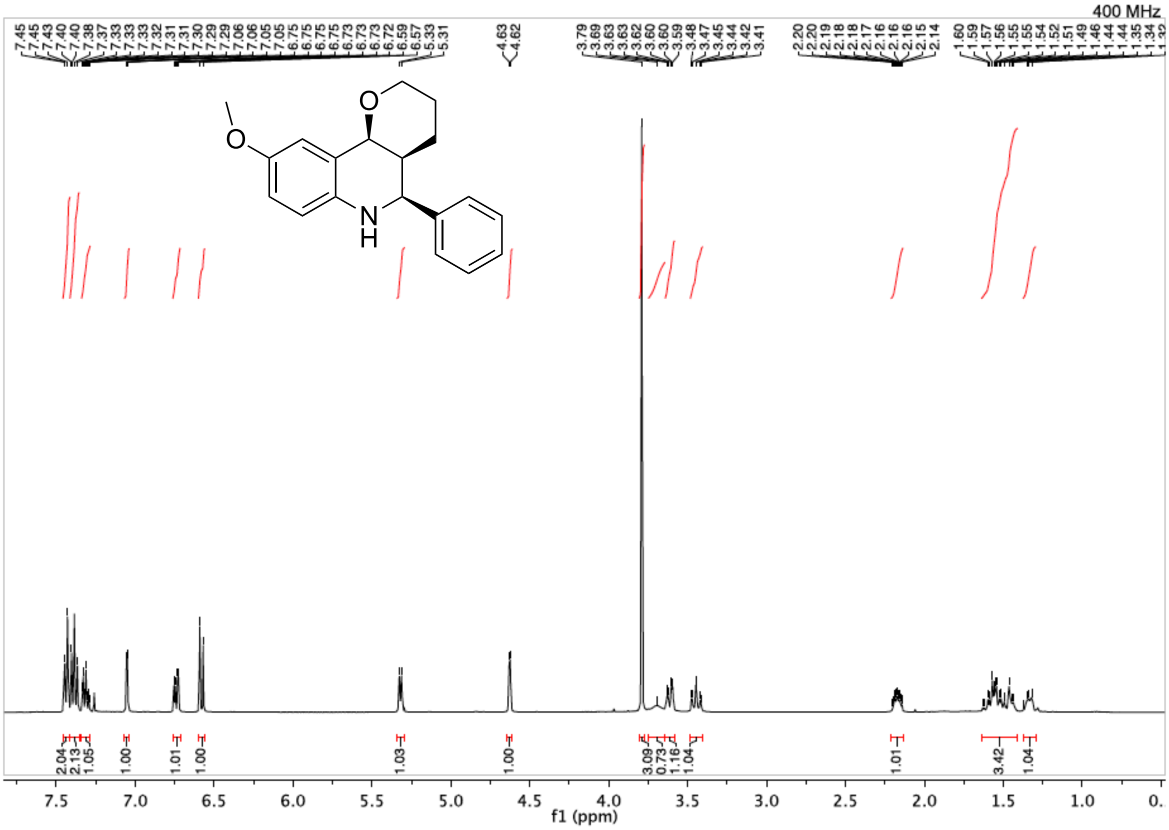

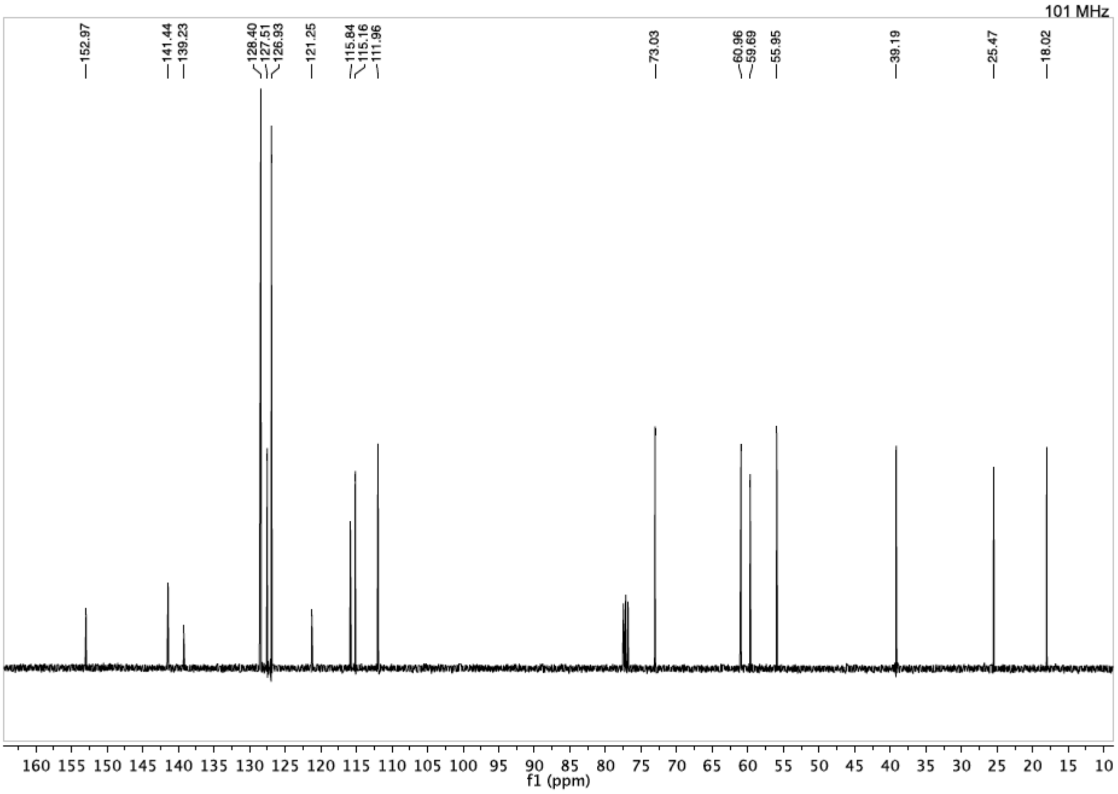

^{1}H and $^{13}C\{^{1}H\}$ NMR spectra in $CDCl_3$

***trans*-9-methoxy-5-phenyl-3,4,4a,5,6,10b-hexahydro-2*H*-pyrano[3,2-*c*]quinoline (8b)**

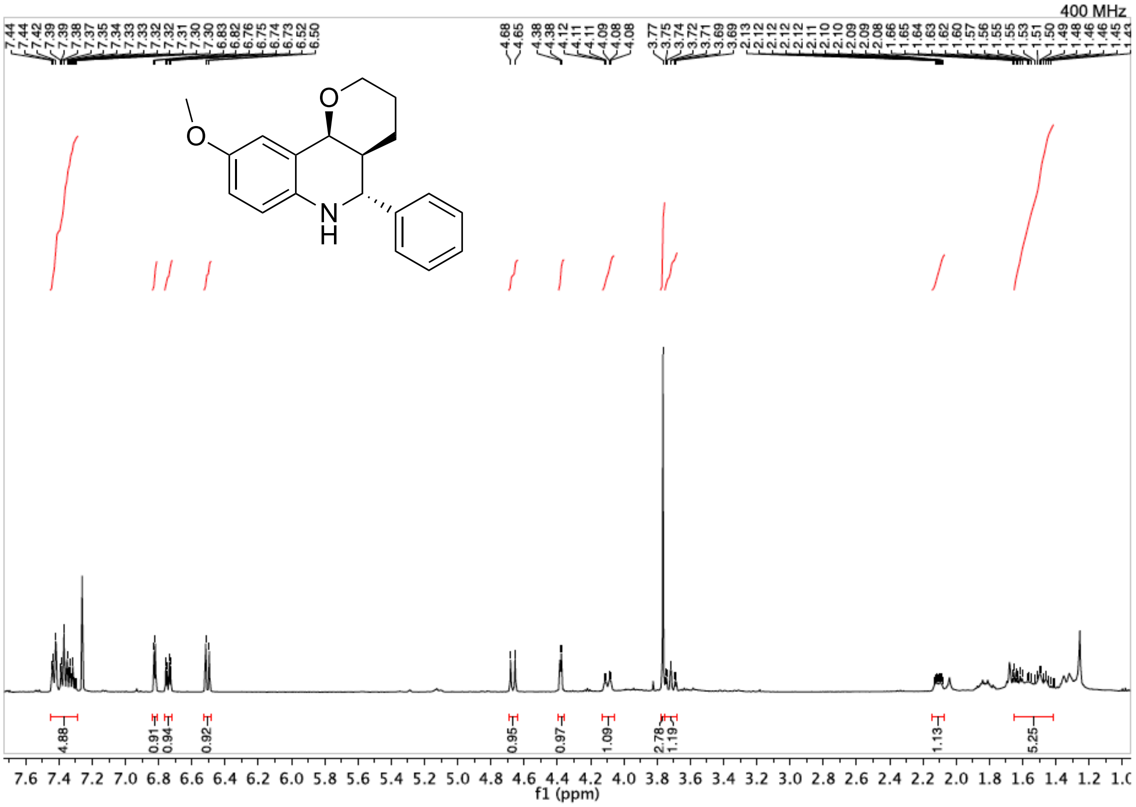

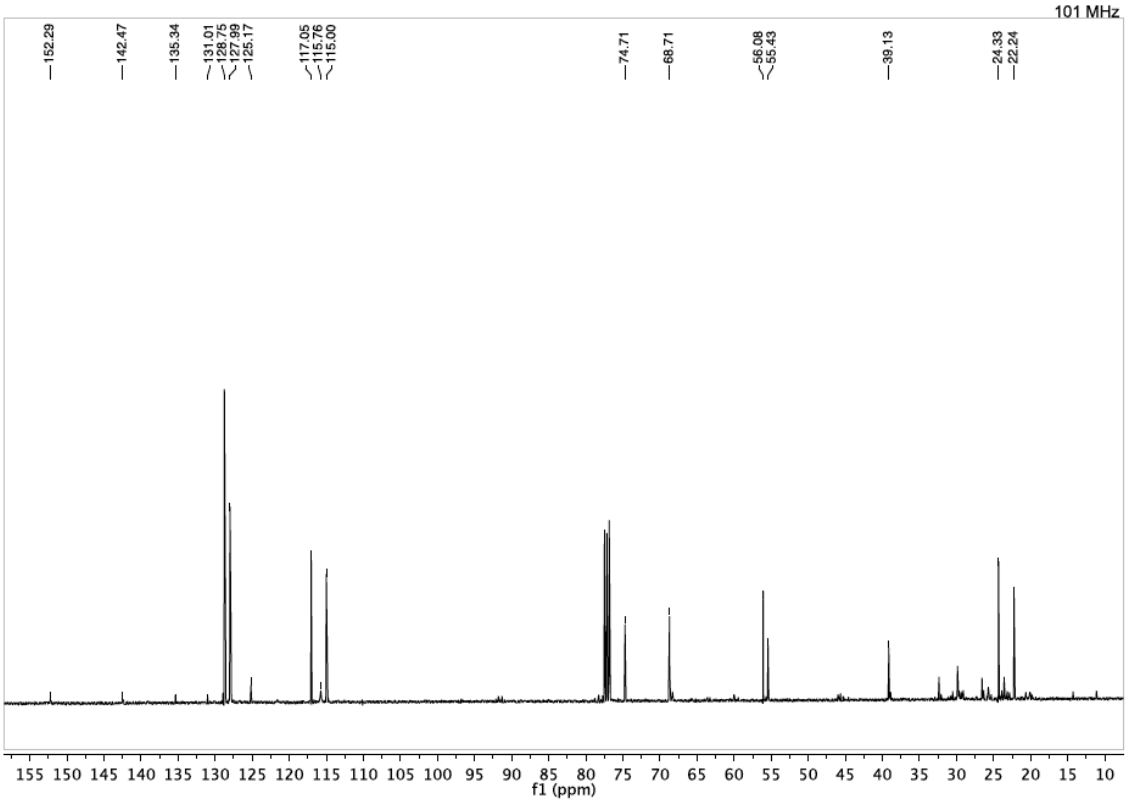

^{1}H and $^{13}C\{^{1}H\}$ NMR spectra in $CDCl_3$

***cis*-5-(4-methoxyphenyl)-9-methyl-3,4,4a,5,6,10b-hexahydro-2*H*-pyrano[3,2-*c*]quinoline (8c)**

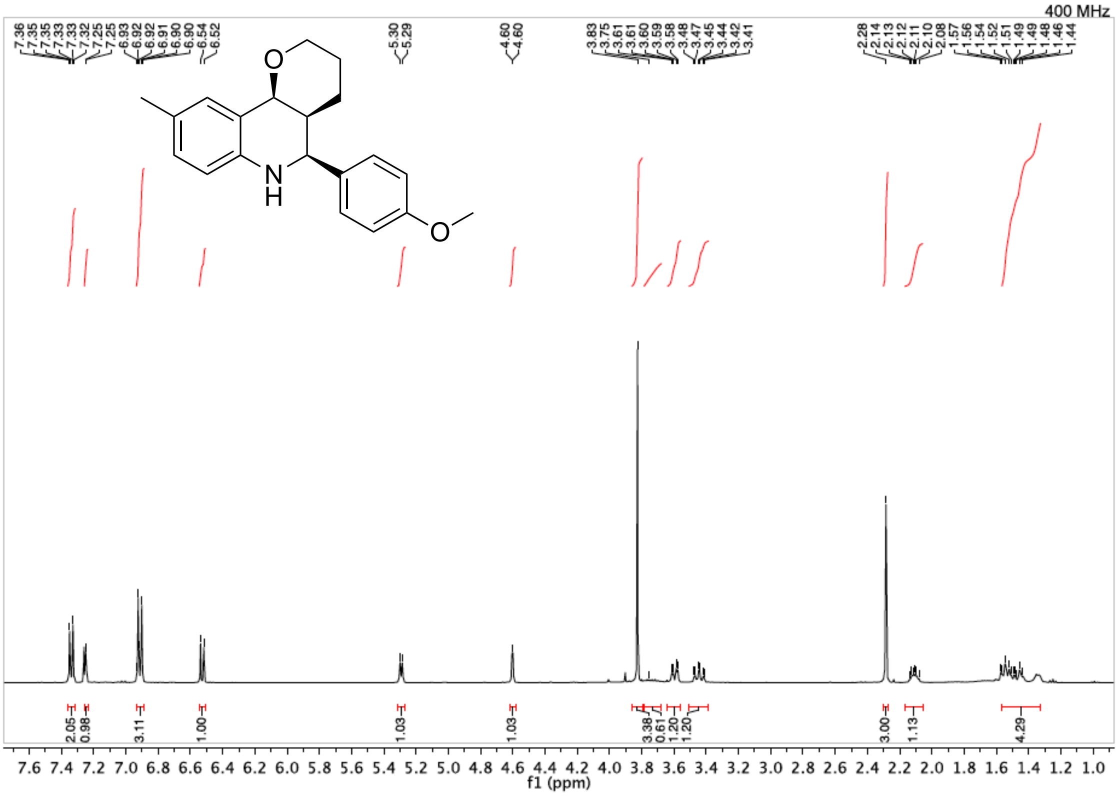

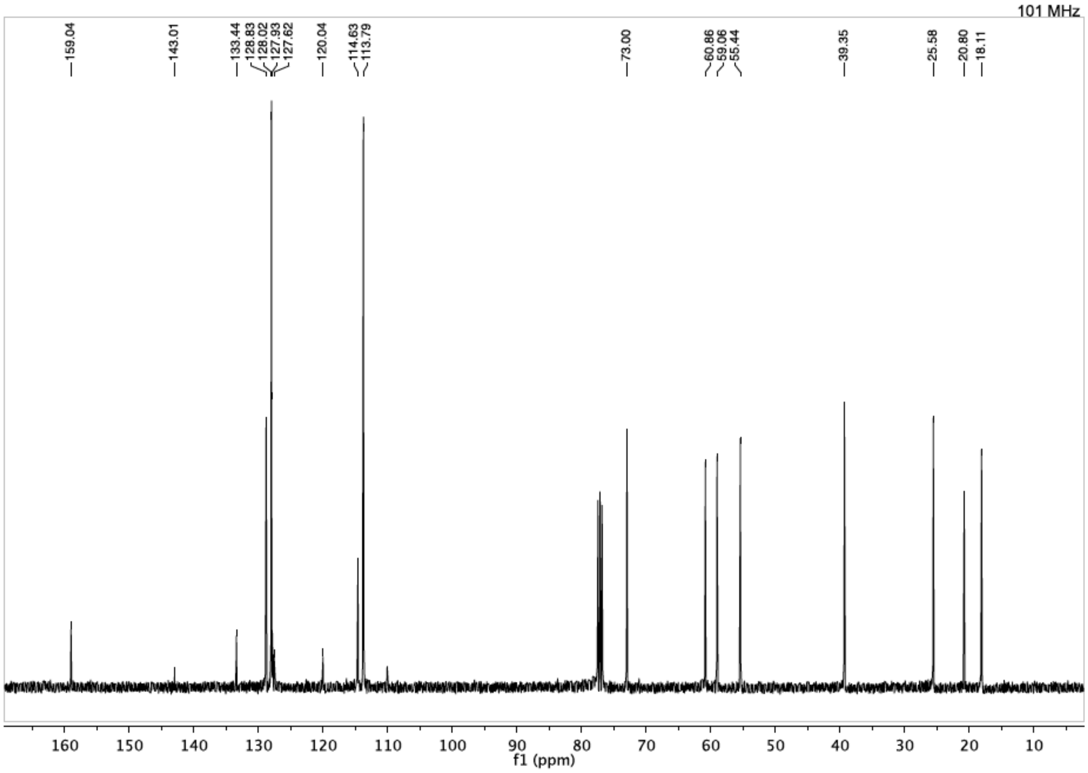

^{1}H and ^{13}C{^{1}H} NMR spectra in $CDCl_3$

***trans*-5-(4-methoxyphenyl)-9-methyl-3,4,4a,5,6,10b-hexahydro-2*H*-pyrano[3,2-*c*]quinoline (8c)**

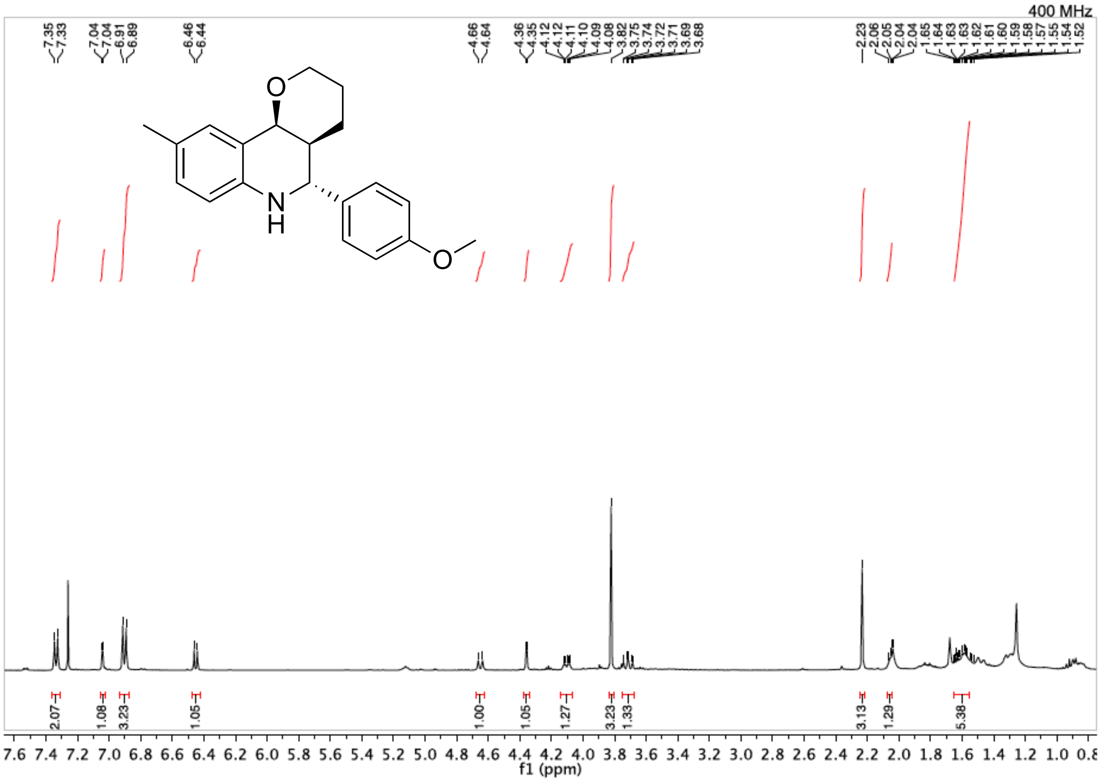

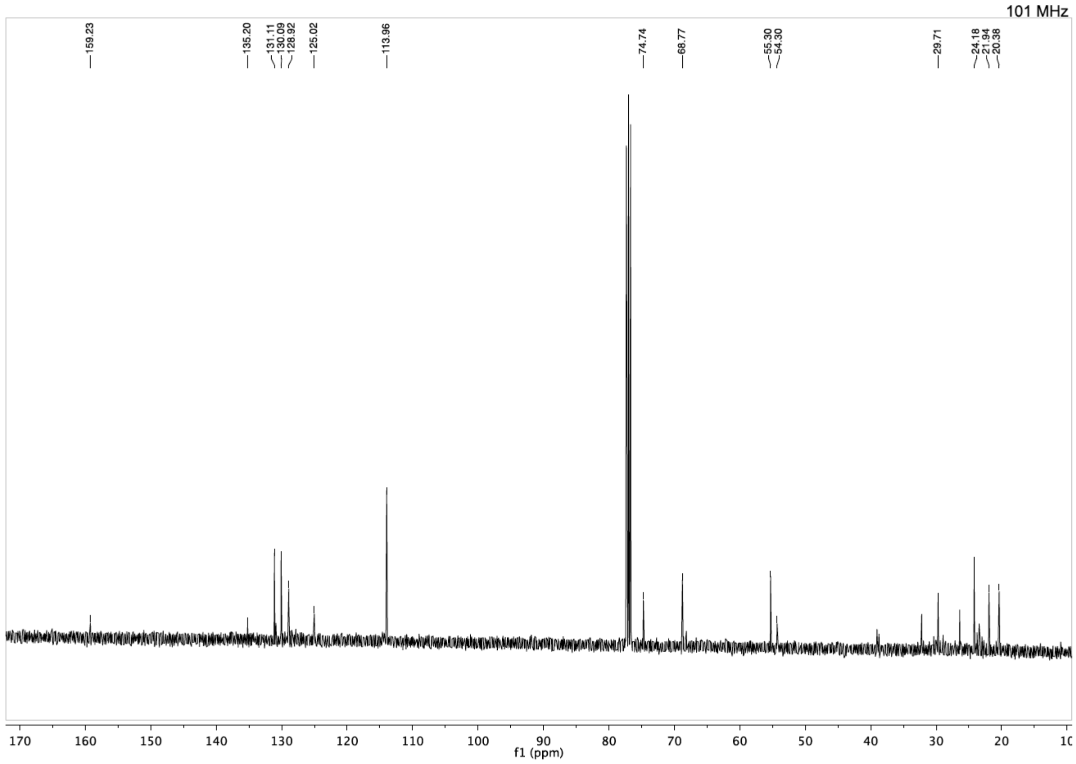

^{1}H and $^{13}C\{^{1}H\}$ NMR spectra in $CDCl_3$

1-benzyl-4-methoxybenzene (*p*-11)

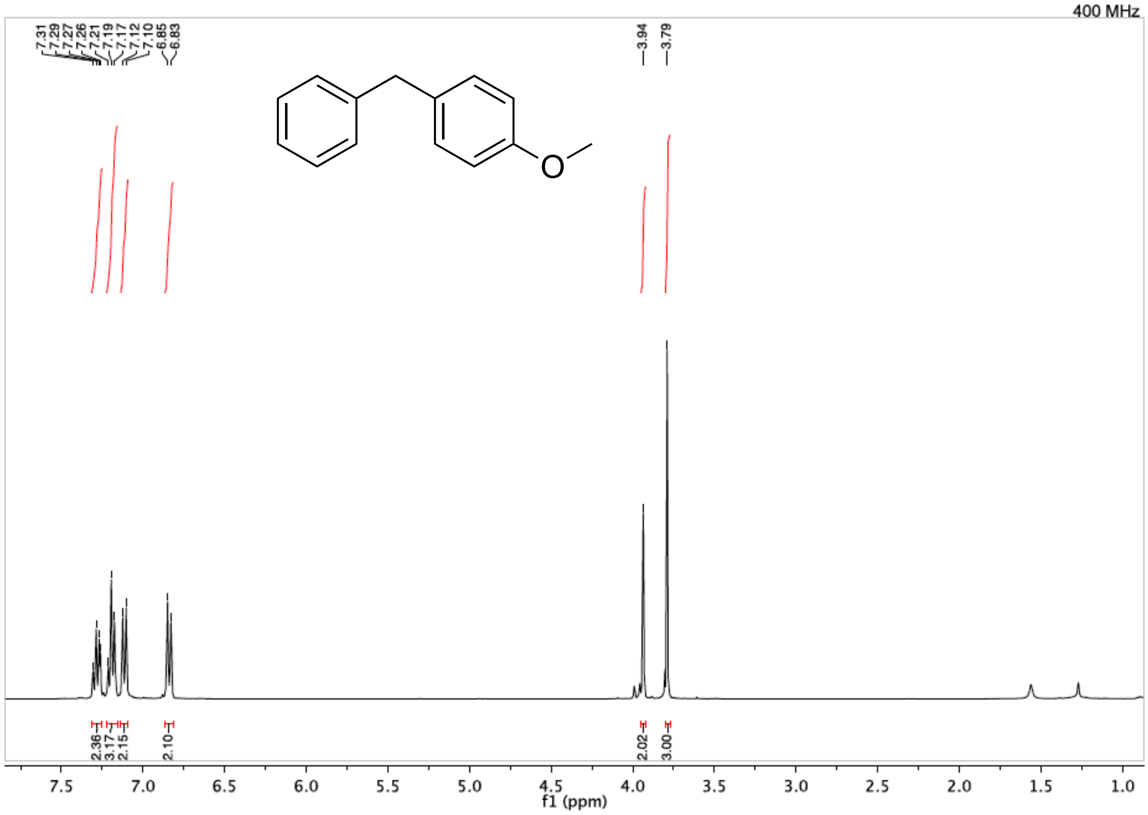

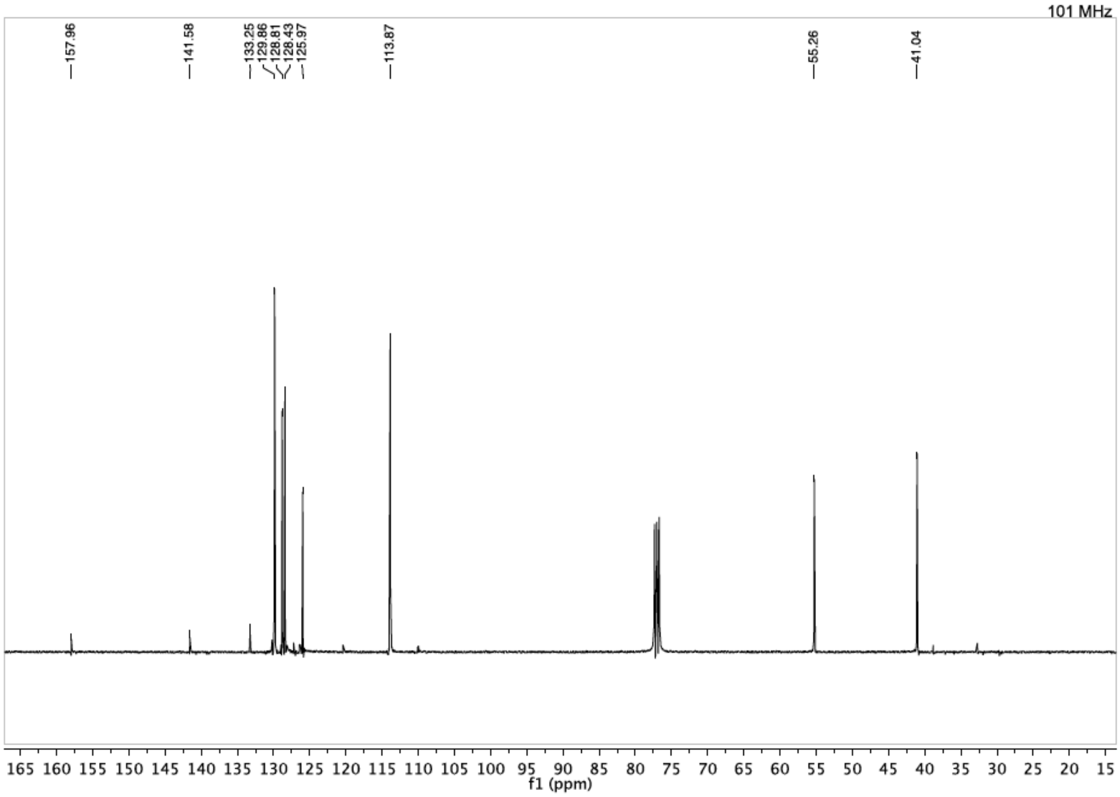

^{1}H and ^{13}C{^{1}H} NMR spectra in $CDCl_3$

1-benzyl-2-methoxybenzene (*o*-11)

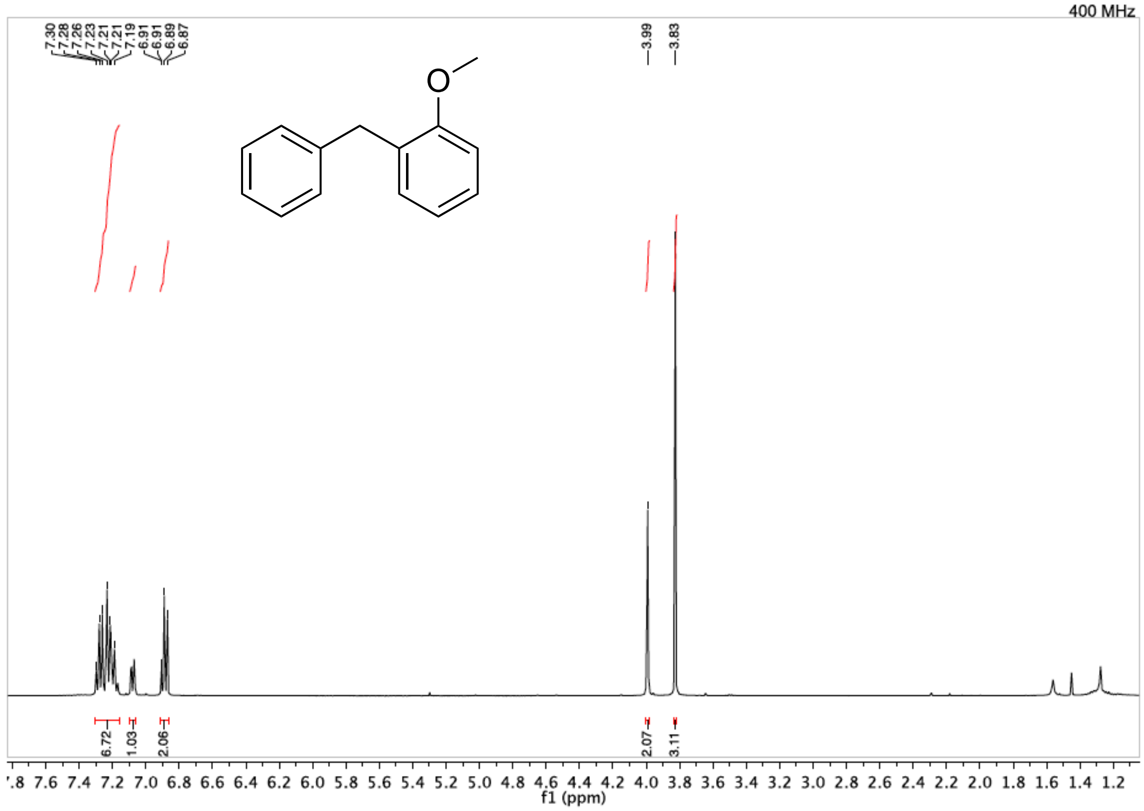

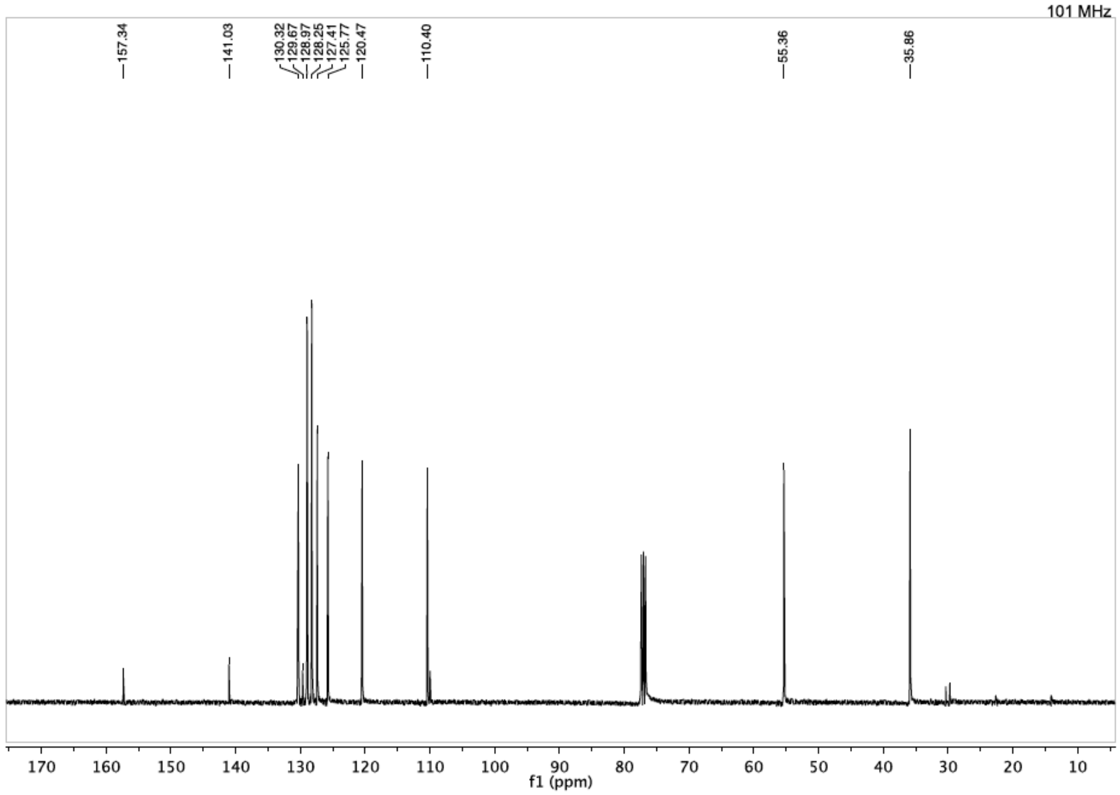

^{1}H and ^{13}C{^{1}H} NMR spectra in $CDCl_3$

9,9-dimethyl-12-phenyl-8,9,10,12-tetrahydro-11*H*-benzo[*a*]xanthen-11-one (13a)

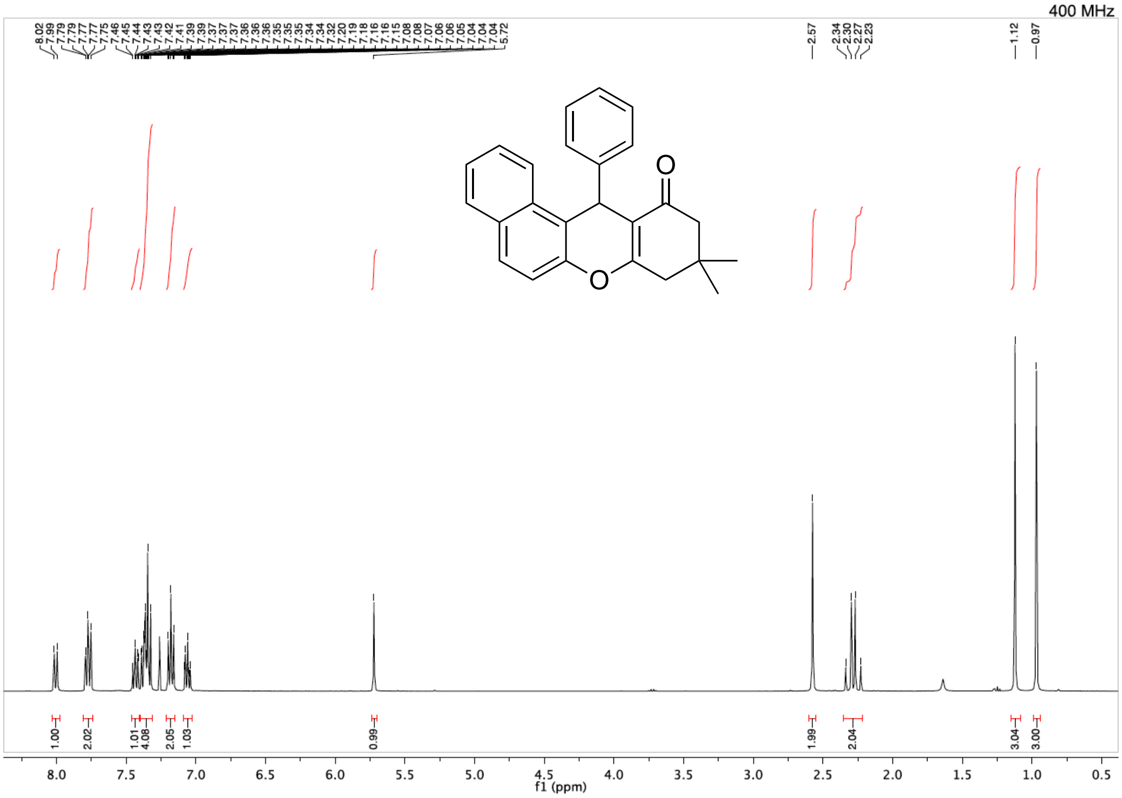

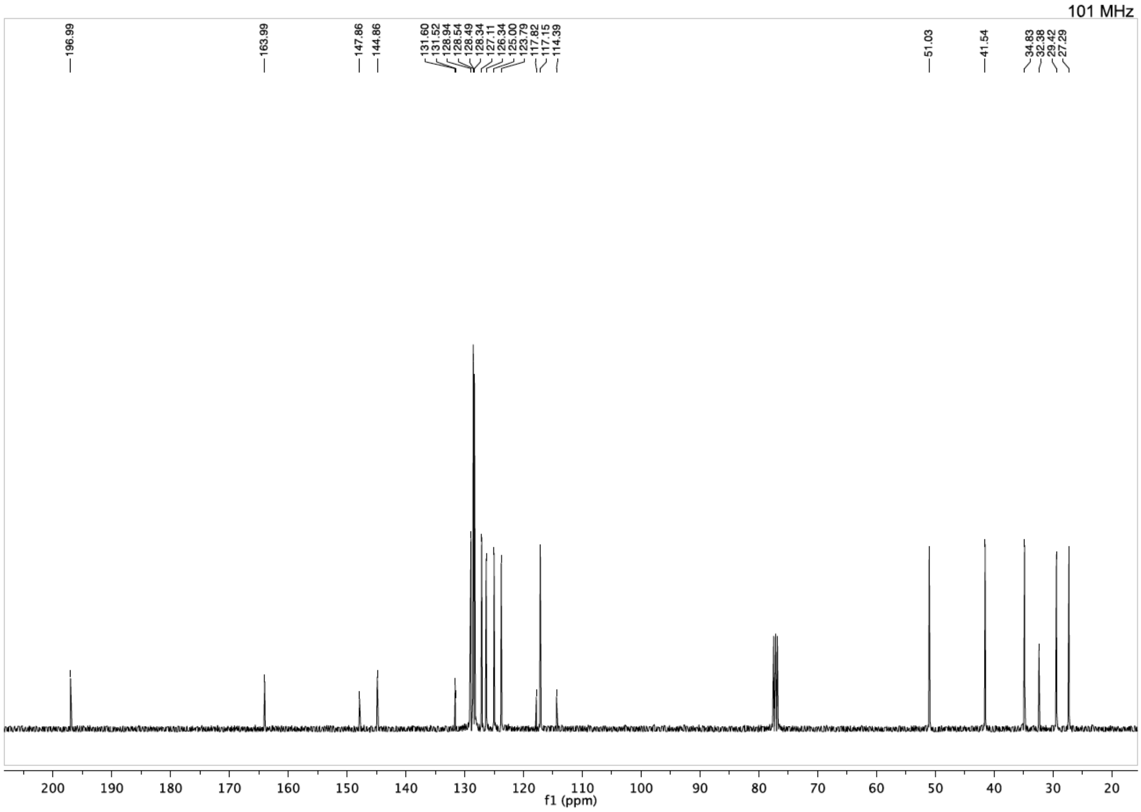

^{1}H and ^{13}C{^{1}H} NMR spectra in $CDCl_3$

12-(4-chlorophenyl)-9,9-dimethyl-8,9,10,12-tetrahydro-11*H*-benzo[*a*]xanthen-11-one (13b)

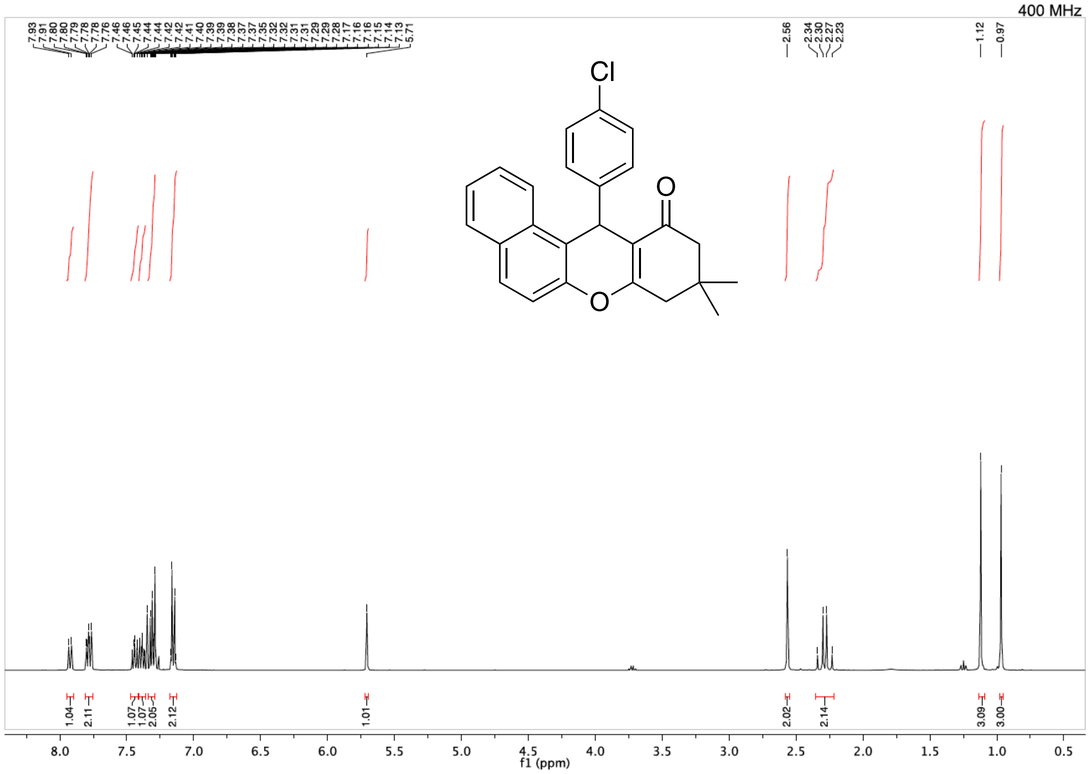

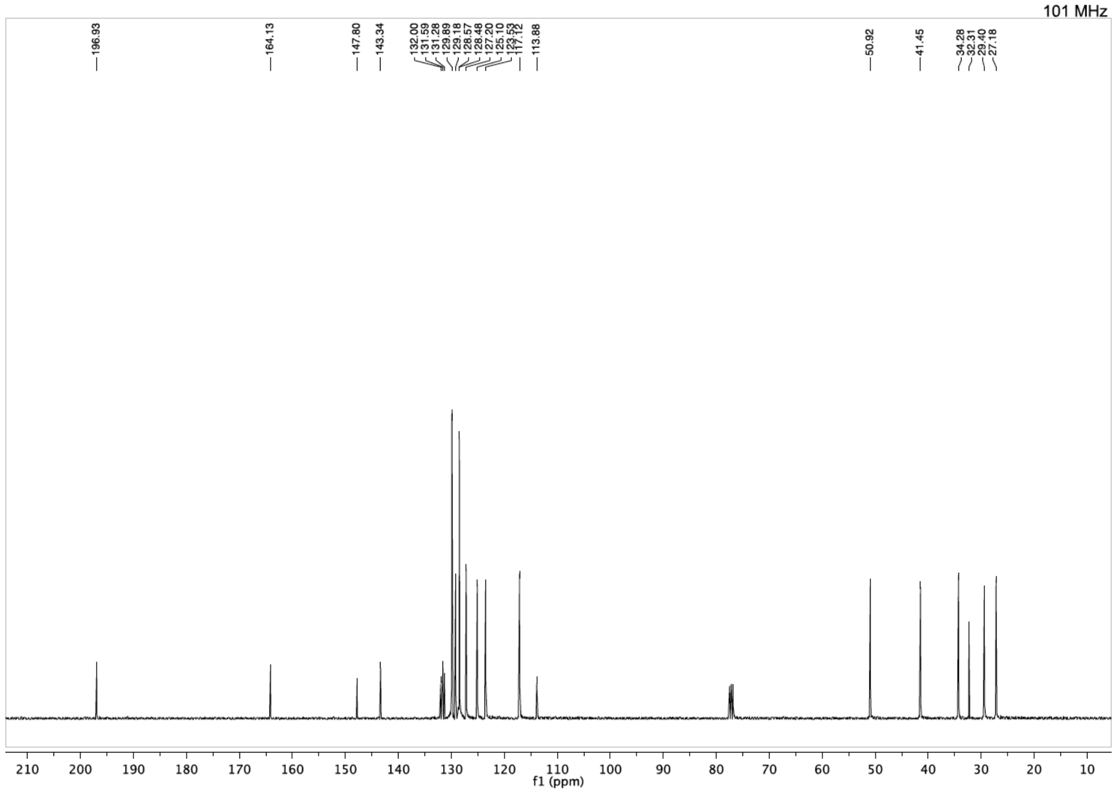

Supplement: Supplementary file 1 — jo1c00932_si_001.pdf [file jo1c00932_si_001.pdf]
